# Synthesis, Morphology, and Particle Size Control of Acidic Aqueous Polyurethane Dispersions

**DOI:** 10.1021/acs.macromol.4c02046

**Published:** 2024-11-14

**Authors:** Ellen J. Quane, Niels Elders, Anna S. Newman, Sophia van Mourik, Neal S. J. Williams, Keimpe J. van den Berg, Anthony J. Ryan, Oleksandr O. Mykhaylyk

**Affiliations:** § School of Mathematical and Physical Sciences, The University of Sheffield, Dainton Building, Brook Hill, Sheffield, South Yorkshire S3 7HF, U.K.; ‡ Department of Resin Technology, Akzo Nobel Car Refinishes BV, Rijksstraatweg 31, Sassenheim 2171 AJ, Netherlands; $ CPI, The Coxon Building, John Walker Road, NETPark, Sedgefield, County Durham TS21 3FE, U.K.

## Abstract

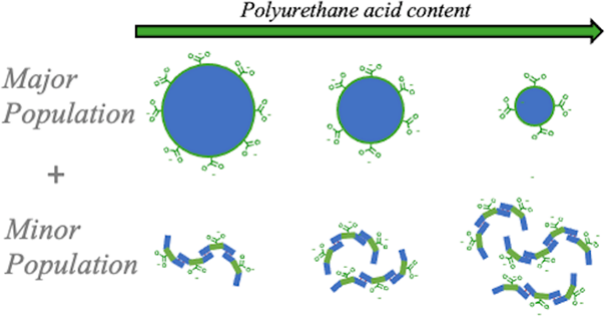

A range of charge-stabilized aqueous polyurethane (PU) dispersions comprising hard segments formed from hydrogenated methylene diphenyl diisocyanate (H_12_MDI) with dimethylolpropionic acid (DMPA) and ethylenediamine, and soft segments of poly(tetramethylene oxide) of different molecular weights are synthesized. Characterization of the dispersions by mass spectrometry, gel permeation chromatography, small-angle X-ray scattering, atomic force microscopy, and infrared spectroscopy shows that they are composed of PUs self-assembled into spherical particles (primary population) and supramolecular structures formed by hydrogen-bonded H_12_MDI and DMPA acid-rich fragments (secondary population). Analysis of the scattering patterns of the dispersions, using a structural model based on conservation of mass, reveals that the proportion of supramolecular structures increases with DMPA content. It is also found that the PU particle radius follows the predictions of the particle surface charge density model, originally developed for acrylic statistical copolymers, and is controlled by hydrophile (DMPA) content in the PU molecules, where an increase in PU acidity results in a decrease in particle size. Moreover, there is a critical fractional coverage of hydrophiles stabilizing the particle surface for a given polyether soft-segment molecular weight, which increases with the polyether molecular weight, confirming that more acid groups are required to stabilize a more hydrophobic composition.

## Introduction

**Figure 1:**
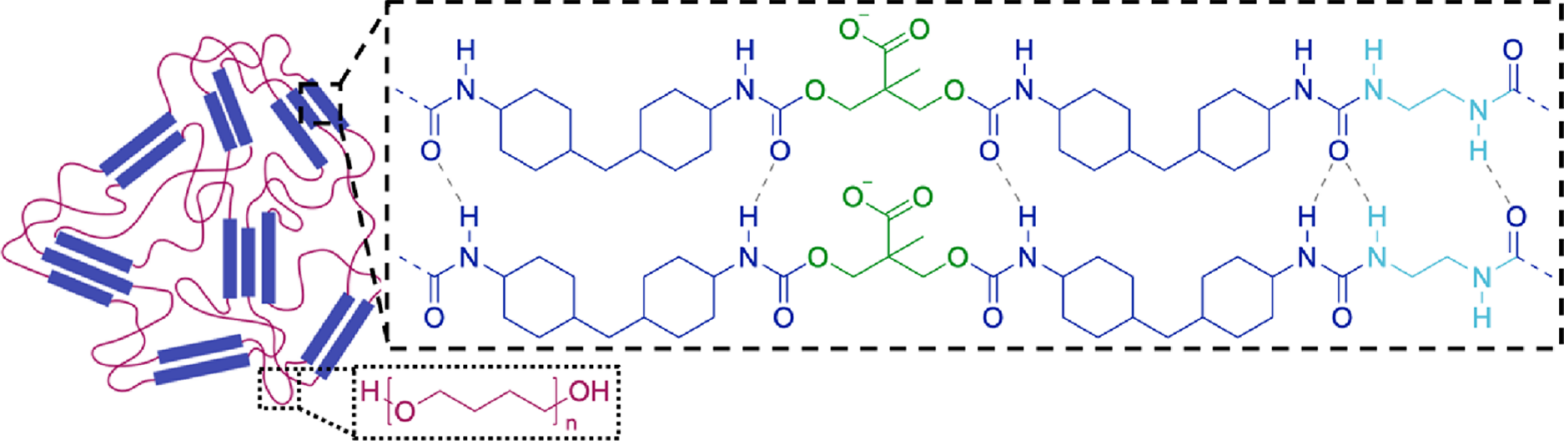
Schematic of polyurethane architecture, based on the polymer composition used in this work, separated into hydrogen-bonding “hard blocks” [comprising diisocyanate (hydrogenated methylene diphenyl diisocyanate, blue), a charge-stabilizing hydrophilic unit (dimethylolpropionic acid, green), and a diamine chain extender (ethylenediamine, turquoise)] and a long-chain polyether soft segment [poly(tetramethylene oxide), purple]. The formation of hydrogen bonds between urethane and urea groups is shown by gray dashed lines.

Polyurethanes (PU) are a versatile and diverse class of polymers that find applications in a wide range of industries including construction, medical, automotive, aeronautical, textiles, and upholstery.^1,2^ Due to the multiplicity of backbone chemistries available, PU can be found as foam in products such as beds and furniture, automotive interiors and shoe soles,^2^ elastomers for biomedical devices,^3,4^ and high-performance adhesives or coatings.^5−7^ The wide range of materials referred to as PU are defined by the reaction of an isocyanate with a functional group containing an active hydrogen, typically an alcohol or amine, to form urethane and urea linkages, respectively.^8−10^ Polymerization to form linear and cross-linked polymers involves the reaction of aliphatic or aromatic diisocyanates with a blend of reactive, di- and polyfunctional monomers. Traditionally, this blend comprises a long-chain polyol and aliphatic chain extender, such as a short-chain diol or diamine. Polyols (such as hydroxyl-terminated polyester,^11−13^ polyether,^11,14−20^ or polycarbonate^16,21^) incorporate soft, flexible segments into the polymer (Figure 1), and the diisocyanate and chain extenders form rigid segments that strongly hydrogen bond through the urethane and urea groups to form hard blocks (Figure 1).^8,9,18^ PU molecules are segmented, multiblock copolymers, and the judicious selection of monomers, combined with microphase separation of the soft segments and the hard blocks (Figure 1), allows PU to develop a broad range of mechanical properties that can be targeted through compositional control to meet specific needs for material performance.^13,19,22−24^

Functional monomer units can also be incorporated into the polymer chain to further elaborate the PU properties. For example, phosphorus-containing polyols have been shown to impart flame-retardancy in coatings,^25^ trifunctional chain extenders allow grafting of dyes for PU textiles,^26^ and *N*-methyldiethanolamine, quaternized with methyl iodide, generates antibacterial textiles resistant to *E. coli*.^15^ Monomers with hydrophilic functionality are widely employed to provide water-based PU dispersions (PUDs) with colloidal stability providing not only high-performance, abrasion-resistant films that are flexible, tough, and solvent resistant,^13,19,22,23^ but also eliminating the need for large volumes of unrecoverable solvent in the PU synthesis. There has, therefore, been an increasing interest in PUDs to replace solvent-borne PU materials in adhesives and coatings, driven by the chemical industry’s efforts to develop environmentally friendly and sustainable products.^13,19,22−24,27^

In principle, incorporation of hydrophilic monomers into the molecular architecture turns PU polymers into amphiphilic statistical copolymers (Figure 1). The hydrophilic units can be nonionic: for example, terminal or lateral poly(ethylene oxide) segments,^10,20^ or ionic, including cationic,^15^ anionic,^20^ and zwitterionic^28^ variants. Nonionic emulsifying groups offer advantages in terms of low viscosities; however, ionic monomers are generally the preferred option for both dispersions with good stability and films with low water sensitivity.^10,15^ Dimethylolpropionic acid (DMPA) is the most commonly used ionic monomer,^29^ which provides charge stability when deprotonated in aqueous solution. The carboxylic acid group also contains an active hydrogen that can react with an isocyanate to generate an amide bond and CO_2_, but the rate of this reaction is slow compared to alcohol and amine, and it does not compete with chain extension. Literature shows that the minimum concentration of DMPA required for stable particles varies depending on the PU composition.^20,30^ Zhang et al. also suggest that the structure of polyester soft segments can contribute to the stability, influencing particle size at low acid contents.^30^ They also show that there is a maximum DMPA content, above which the PU polymer becomes fully soluble, preventing the formation of a dispersion. It is well-established that varying the DMPA content, between the thresholds for colloid stability and complete solubility, controls both the interfacial tension and repulsion between particles,^11,14^ as well as the particle size in PU dispersions.^19,20,30,31^ This inherent relationship between particle size and acid content also brings about a correlation between particle size and other properties such as solution viscosity,^13,31,32^ stability,^30,33^ and adhesion.^19^ In unison with the findings for PU dispersions, similarly amphiphilic systems of statistical acrylic copolymers demonstrate that the particle size can be predicted based on the mole fraction of hydrophilic comonomers.^34,35^ Therein a particle surface charge (PSC) model was proposed that identifies critical surface area coverage by hydrophilic monomers (SA_frac_) as the key factor controlling the particle size. It was also found that a greater surface area coverage of hydrophiles is necessary for comonomers with higher hydrophobicity.^35^

Although there are a variety of recognized PUD synthesis methods, the majority are made by either the acetone method or prepolymer mixing method.^10,24^ Both methods begin with the production of short isocyanate-capped PU fragments, known as prepolymers, by the reaction of diols with an excess of diisocyanate. For the acetone method, an organic solvent is used for the chain extension step, where these fragments are linked by small molecule diamines or diols, to give the final high-molecular-weight PU chains. These PU polymers are then dispersed in water, and the solvent is removed. This method is known to give highly repeatable results and good control of the products.^10,13,27^ In contrast, the steps are reversed in the prepolymer mixing method, such that the prepolymer fragments are dispersed in water prior to chain extension, eliminating the need for large volumes of organic solvents to control viscosity.^10,12,13,27,36^ Despite the environmental and economic advantages of reduced solvent usage in this method, there is a penalty in terms of the quality of the resulting products. The production of poorer quality dispersions, which require higher acid contents for stable particles compared to the acetone process, is often attributed to chain extension in heterogeneous media.^10,13,20,27^ However, there is still no clear understanding of the chemical reactions and structural morphologies formed during the prepolymer mixing method and why they give rise to poorer dispersions and films.

Structural characterization of PU materials is usually focused on the morphology of microphase separation^37^ where small-angle scattering (SAS) techniques are very informative,^37−47^ providing extensive information such as hard block thickness, diffuse boundary thickness, surface-to-volume ratios, phase purity, and degree of crystallinity.^41−43^ SAS can also be very informative on particle morphologies in solution,^48^ yet only a few studies using SAS for extensive characterization of PU particles in aqueous dispersion have been published, reporting interesting structural features that are assessable only by nondestructive scattering techniques.^20,49^ Bolze et al. used a small-angle X-ray scattering (SAXS) contrast variation technique to demonstrate phase separation of PU within the individual particles in an aqueous dispersion,^49^ whereas Satguru et al., by means of small-angle neutron scattering (SANS), indicated that water pockets could be present in the PU particles dispersed in water.^20^

The sparse literature available indicates that SAS can be very effective for the structural characterization of PUDs. Given that PUs are a chemically diverse class of polymers and a broad range of PUD compositions is possible, it is paramount to establish particle structure–composition relationships that could be used as a predictive tool for the formation of stable PU particles in aqueous dispersions. This study comprehensively investigates the impact of molecular composition (polyether molecular weight, hard block content, and carboxyl group concentration) on the self-assembly of PU in water. SAXS is employed to probe PU particle composition, size, and shape, as well as to critically examine evidence for microphase separation and the presence of water. Step growth polymerization, including PU synthesis, is a stochastic process producing a most probable distribution of polymeric species.^50^ In particular, when the degree of polymerization is kept low, such as in the production of PU prepolymers, the product will inevitably contain short, isolated hard block sequences that are not connected to a soft-segment moiety, including highly acidic fragments of alternating DMPA and diisocyanate, which are not usually considered important. In this study, indeed, these products of PU synthesis by the prepolymer mixing method are found to be present in the PU dispersions using matrix-assisted laser desorption/ionization (MALDI) and gel permeation chromatography (GPC). It is further demonstrated by SAXS analysis that these water-soluble, hard-segment sequences hydrogen bond into supramolecular structures and form a minority second population of self-assembled molecules with their concentration depending on the PU composition, in particular, acid content. These results are further corroborated by atomic force microscopy (AFM) and Fourier-transform infrared spectroscopy (FTIR). Furthermore, it is shown that the size of spherical particles in the primary population, comprising high-molecular-weight PU with soft and hard segments, is controlled by the acid content and soft-segment length, confirming the general rule for self-assembly of amphiphilic statistical copolymers initially found for acrylic systems.^34,35^

## Materials and Methods

The PU polymers in the studied PUDs are composed of hydrogenated methylene diphenyl diisocyanate (H_12_MDI), DMPA and ethylenediamine forming hard blocks (HBs), and poly(tetramethylene oxide) (PTMO) corresponding to soft segments (SSs) (Figure 1, Table 1, and Table S1, see the Supporting Information). Samples were produced using three molecular weights of PTMO, at three hard block contents (Table 1). To highlight the chemical composition of the samples, the HB content and SS number-average molecular weight (*M*_n_) are given in the sample names, e.g., HB50_SS1000 refers to a PUD with 50 wt % hard block and soft segment of *M*_n_ = 1000 g mol^–1^ (Table 1). All PU prepolymers were designed at an NCO/OH molar ratio of 1.5 and made following the prepolymer method in a four-step reaction: (1) prepolymer synthesis, (2) deprotonation, (3) emulsification, and (4) chain extension. Synthesis details, material ratios, and PTMO molecular weights are given in the Supporting Information (Table S1).

**Table 1:** Composition of the Synthesized PUDs

sample	PU hard block content, wt.%	soft segment *M*_n_, g mol^–1^^b^	COOH Content, wt.%
HB50_SS650	50	650	1.52
HB60_SS650	60	650	2.83
HB70_SS650	70	650	4.15
HB50_SS1000	50	1000	2.40
HB60_SS1000	60	1000	3.57
HB70_SS1000	70	1000	4.72
HB50_SS2000	50	2000	3.29
HB60_SS2000	60	2000	4.23
HB70_SS2000	70	2000	5.22
H_12_MDI_DMPA^a^	100		6.84

a Refers to the product of the 2:1 molar ratio reaction of H_12_MDI:DMPA.

b Dispersity of the commercial PTMO diols used for the PU soft segment was about 2.0.

PUD samples were primarily characterized by SAXS, providing information about internal morphology and particle sizes. Scattering patterns were recorded over a *q* range of 0.007 Å^–1^ ≤ *q* ≤ 0.2 Å^–1^, where *q* is the scattering vector length defined by
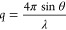
1where θ is half of the scattering angle and λ is the wavelength of the X-ray radiation. The total scattered intensity from a dispersion of multiple populations of scattering objects, including particles such as PU, can be expressed in a general form as
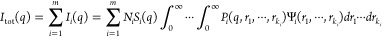
2where *m* is the number of populations; *I*_*i*_(*q*), *P*_*i*_(*q*, *r*_1_, ···, *r*_*k*_*i*__), and Ψ_*i*_(*r*_1_, ···, *r*_*k*_*i*__) are the intensity of scattering, the form factor [including volume and excess scattering length density (SLD) of the objects] defined by a *k_i_* number of parameters, and the multivariate distribution function normalized as
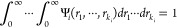
3of *i*^th^ population, respectively. *N*_*i*_ is the number density of the *i*^th^ population expressed as
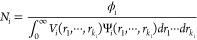
4where ϕ_*i*_ and *V*_*i*_(*r*_1_, ···, *r*_*k*_*i*__) are the volume fraction and scattering object volume of the population, respectively. For the sake of simplicity, a local monodisperse approximation is assumed in eq 2 that only objects belonging to the same population interact with each other and any particle of a given size in the population is surrounded by particles of the same size,^48^ which is expressed via an effective structure factor term *S*_*i*_(*q*). For a single population of spherical particles (*m* = 1), where dispersity of only a single parameter such as particles' radius *r* is considered, eq 2 can be rewritten as
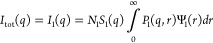
5

The scattering form factor of spherical particles in this equation is defined as
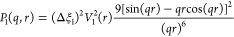
6where Δξ_1_ is the SLD contrast between the particles and solvent and  is the spherical particle volume.

The mass density of the PU polymer in each of the PUDs studied was found from solution density measurements (Figures S2 and S3 and Table S2). These values were used for the calculations of SLDs (eqs S2 and S3).

GPC was used to obtain molecular weight distributions for the PU prepolymer fragments, and MALDI time-of-flight spectra were collected on final aqueous PUDs. Additionally, FTIR was used to probe the hydrogen bonding that is prevalent in PU hard blocks (Figure 1). AFM was employed to verify the particle morphology. Spin-dried films from selected dilute dispersions were prepared to maintain the morphology of the particles in their wet state. Further details on the characterization of the products can be found in the Supporting Information.

## Results and Discussion

**Figure 2:**
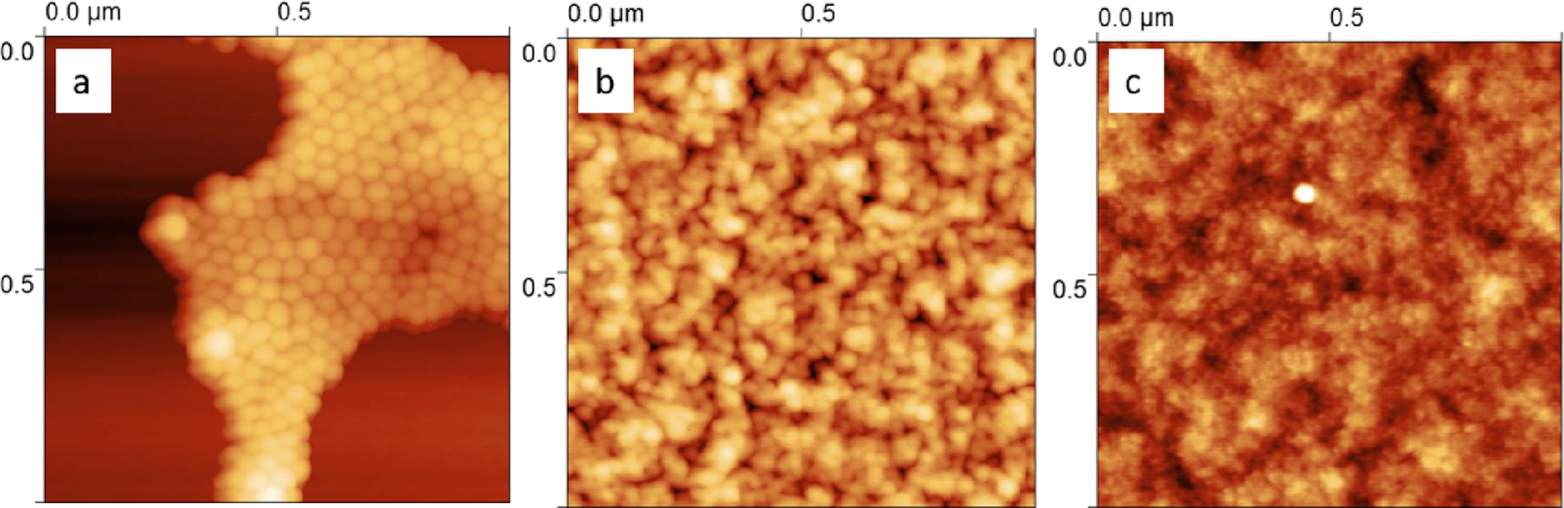
Representative AFM images of spin-coated aqueous PU dispersions composed of 50 wt % hard block and polyether of *M*_n_ (a) 650 g mol^–1^, (b) 1000 g mol^–1^, and (c) 2000 g mol^–1^.

**Figure 3:**
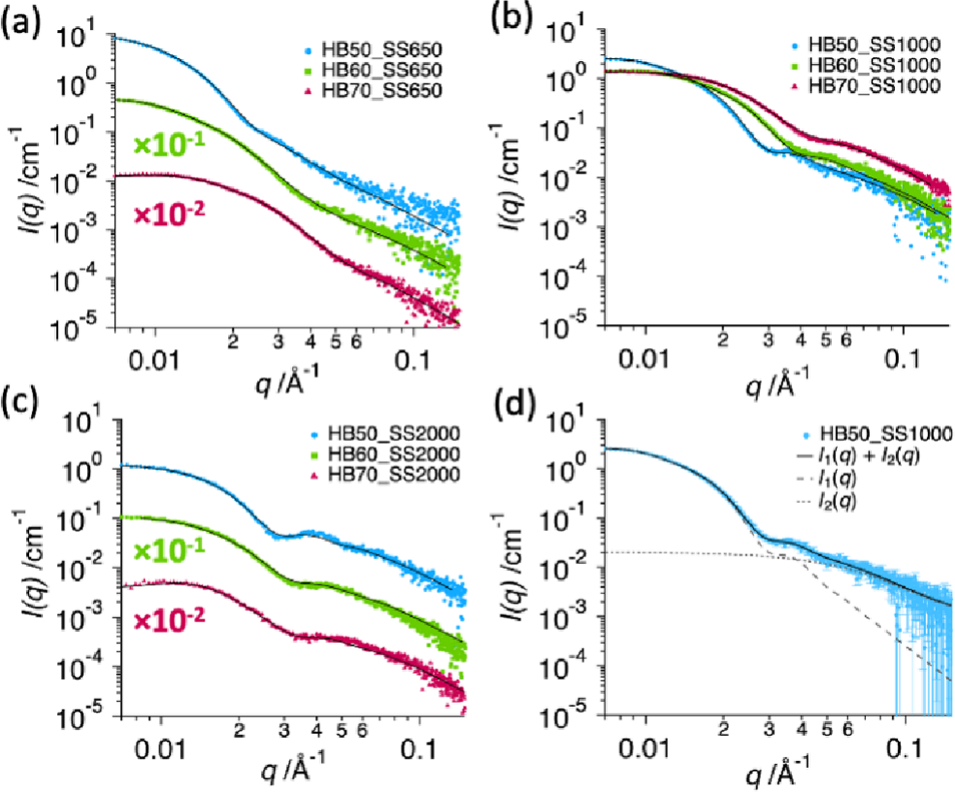
SAXS profiles for 1 wt % aqueous dispersions of PU, composed of soft segment of *M*_n_: (a) 650 g mol^–1^, (b) 1000 g mol^–1^, and (c) 2000 g mol^–1^, with hard block contents of 50 wt.% (blue circles), 60 wt.% (green squares), and 70 wt.% (red triangles). Some of the profiles are offset by the indicated multiplication factor to avoid their overlap. Solid curves show fitting of the SAXS model. Plot (d) shows, for a representative HB50_SS1000 PUD, the decomposition of the SAXS model fitting curve into scattering signals originating from two populations: spherical PU particles, *I*_1_(*q*) (dotted curve), and supramolecular PU structure, *I*_2_(*q*) (dashed curve).

It is well-established that charged PU molecules self-assemble as spherical particles in aqueous dispersions.^13,20,29−31,36,49^ Indeed, representative AFM images of the spin-coated PUDs synthesized in this work indicate the presence of spherical particles (Figure 2), especially for PU samples composed of the smallest SS molecular weight, 650 g mol^–1^ (Figure 2a). In addition, SAXS profiles of the PUDs (Figure 3) can be fitted reasonably well at low *q* values (*q* < 0.03 Å^–1^) using a single population of spherical PU particles in aqueous dispersion (eq 5) [Figure 3d, *I*_1_(*q*)]. Despite the low PU concentration used for this data collection, some of the SAXS profiles exhibit a weak, broad peak at *q* < 0.02 Å^–1^, indicating interactions between neighboring particles forming short-range structural order across the sample.

This is likely to be caused by repulsion between acidic moieties present on the surface of particles, and in accordance with this, the most acidic sample (HB70_SS2000) shows the most noticeable structure peak (Figure 3c, red triangles). This phenomenon is well-established for charged nanoparticles in dispersion.^34,35,51,52^ It was demonstrated previously^35^ that, while the Hayter–Penfold approximation for a charged sphere structure factor^53^ is a physically more appropriate expression for describing particles undergoing charge repulsion, the less parametrized hard-sphere structure factor, solved with the Percus–Yevick closure relation,^54^ provides a sufficient analytical expression for modeling the structure peak in the SAXS data analysis. This is commonly used in scattering analysis for counting the effects of particle interactions.^55^ Thus, the hard-sphere structure factor was chosen for *S*_1_(*q*) (eqs 2 and 5). The parameters of interest in this study—namely, particle radius and its dispersity—are contained in the form factor and are essentially independent of the structure factor expression selected.

The scattering curve of the spherical particle model demonstrates a significant deviation from the experimental data at *q* > 0.03 Å^–1^ [Figure 3d, *I*_1_(*q*)]. This indicates that there is an additional scattering signal contributing to the total intensity at higher *q* values. The diffuse scattering at high *q* values was observed for PU of similar composition before.^49^ This was previously attributed to a phase-separated structure formed within the PU particles by alternating hard and soft-segment regions. Based on this interpretation, a change in the HB content and/or SS molecular weight would directly control the phase separation of these components in the PU particles. As such, if the excess scattering feature was assigned to the phase separation, it would be most intense at 50 wt % HB content. However, SAXS profiles of the PUDs show the opposite trend in the region of the excess scattering, exhibiting a minimum intensity contribution at 50 wt.% of HB, and a maximum at 70 wt.% of HB (Figure 3b and Figure S1). This analysis suggests that phase separation within the particles cannot be the source of the observed excess scattering. However, the possibility of phase separation occurring in the PU particles cannot be completely ruled out, as it could produce a weak scattering signal in this high *q* region that does not significantly influence the overall SAXS profile.

There was a report that PU particles in aqueous dispersion could have “an open, water-swollen” structure.^20^ Based on SANS results, it was proposed that the PU particles contained water-rich areas, although it was acknowledged that the model applied to the SANS data could not provide unambiguous information about the particle morphology. Nevertheless, the presence of water inside the PU particles has since been corroborated by self-consistent field theory calculations.^56^ Indeed, water molecules can be associated with any acidic moieties trapped in the interior of particles. The water-rich areas, or pockets, located inside of PU particles should produce SLD fluctuations, causing excess X-ray (or neutron) scattering, related to their form factor. In this respect, a “blob” model, proposed for describing SLD fluctuations in micelles resulting from solvent swelling,^57^ can be adapted (eqs S4–S7) to analyze the PUD scattering profiles to establish the presence of water pockets in the PU particles. However, the application of this model for the SAXS data analysis did not yield suitable parameters to produce satisfactory fitting curves to the PUD scattering profiles (Figure S4). Thus, neither HB-SS phase separation nor the water pockets within the PU particles, suggested in the literature as potential sources of internal particle inhomogeneities that could cause excess scattering signal observed at *q* > 0.03 Å^–1^, adequately describe the observed PUD scattering profiles (Figure 3).

Preliminary SAXS analysis has indicated that the excess scattering at high *q* values can be fitted satisfactorily using an analytical expression for the form factor of a Gaussian chain, corresponding to polymers in solution [Figure 3d, *I*_2_(*q*)]:^58^
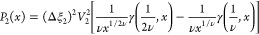
7where *V*_2_ is the volume of a single chain (eq S8), Δξ_2_ is the SLD contrast between the polymer and solvent (eq S2), ν is the excluded volume parameter related to the solvent–polymer interactions, and γ is the incomplete Gamma function:
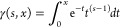
8

Variable *x* in eqs 7 and 8 represents *q* and the radius of gyration of the coiled polymer, *R*_g_:
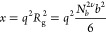
9where *N*_b_ is the number of Kuhn lengths *b* in the fully extended polymer chain. Thus, this SAXS analysis indicates that the PUDs may contain a second population of scattering objects, such as solubilized polymer molecules. It can be considered that due to the high proportion of acid in the polymer composition and the statistical nature of the polymer architecture, it is possible that some polymer chains and/or their short fragments may be highly charged and prefer the water phase rather than the formation of particles. To test this hypothesis, MALDI and GPC analyses were employed to look for these molecules.

**Figure 4:**
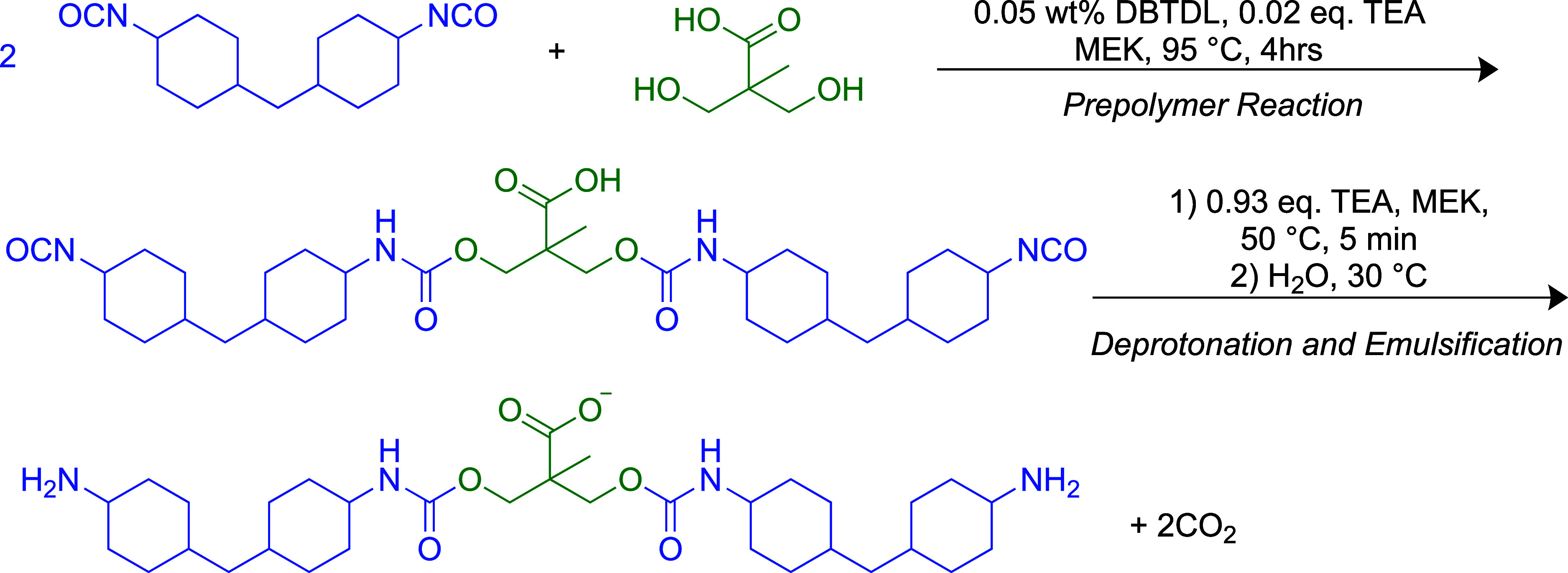
Schematic of the formation of amine-capped H_12_MDI-DMPA-H_12_MDI molecules during the production of PUD.

Representative MALDI spectra were obtained for samples containing PTMO of *M*_n_ = 2000 g mol^–1^, as the SAXS profiles for these PUs exhibit a strong signal from the detected excess scattering (Figure 3c) deviating from the expected intensity gradient of −4 for spherical particles with sharp interfaces at high *q* values [Figure 3d, *I*_1_(*q*)]. MALDI spectra, taken in negative mode, all show a significant peak corresponding to a fragment with a molecular weight of 604 g mol^–1^ (Figure S5). This can be attributed to the H_12_MDI-DMPA-H_12_MDI molecule, with amine caps and a deprotonated acid group (Figure 4).

Because of the excess of H_12_MDI in the prepolymer reaction, it is statistically predicted^50^ that a significant fraction of the PU would form such H_12_MDI-DMPA-H_12_MDI fragments. As the prepolymer mixing method has been used, the isocyanate groups have been converted to amines due to the reaction of isocyanate with the large excess of water upon emulsification. Although the reaction of isocyanate with the chain extending amine is about 100 times faster than the reaction with water, the volume of water molecules compared to the chain extender (approximately 1:300 chain extender:water molar ratio) is such that, statistically, the reaction with water can be competitive, forming the amine-capped fragments. Due to their enhanced hydrophilicity, it is probable that these acidic fragments would form a second, separate population in the PU dispersions.

**Figure 5:**
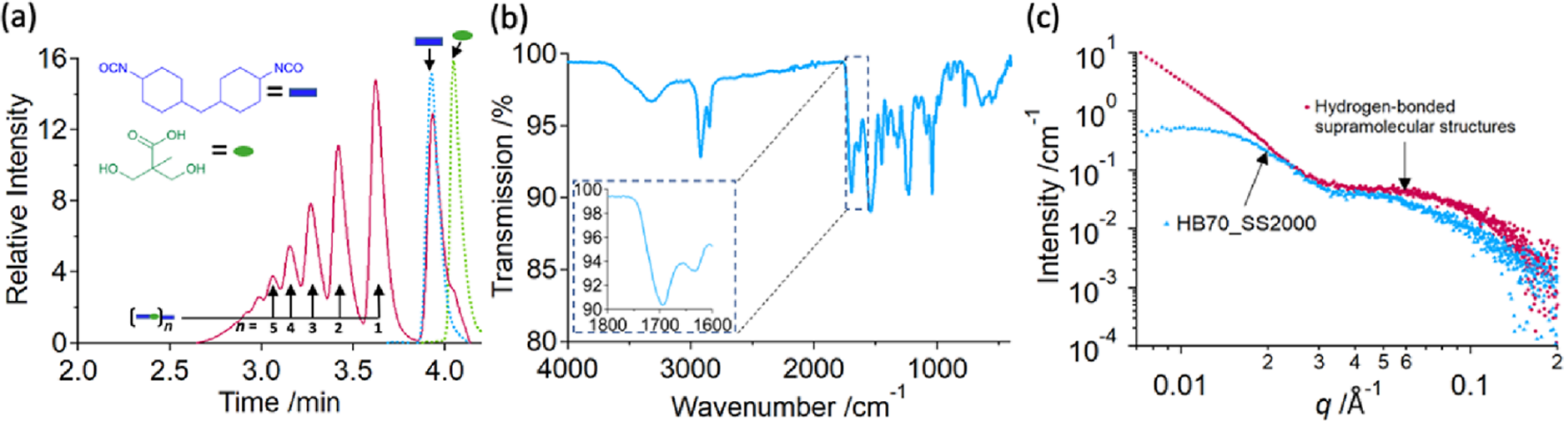
(a) GPC spectra for the product of prepolymer synthesis with an H_12_MDI:DMPA molecular ratio of 2:1 (H_12_MDI_DMPA) (red line), pure H_12_MDI (blue dashed line), and pure DMPA (green dashed line). The origin of peaks is labeled by blue rectangles and green ovals, representing H_12_MDI and DMPA, respectively. “*n*” describes the composition of the H_12_MDI-DMPA fragments, which comprise *n* DMPA and (*n* + 1) H_12_MDI. (b) FTIR spectra of a dried H_12_MDI_DMPA film. The inset shows the region of C=O bond stretching (∼1700 cm^–1^) and N–H bond bending (∼1630 cm^–1^) of urethane. (c) SAXS profile of 1.5 wt % aqueous solution of H_12_MDI_DMPA (red) compared to a representative PUD sample, 1 wt % aqueous dispersion of HB70_SS2000, (blue).

To further underpin the MALDI findings, the H_12_MDI-DMPA-H_12_MDI fragments were targeted by reacting a 2:1 molar ratio of H_12_MDI:DMPA (Table 1). At the end of the reaction, a small sample was quenched with ethanol for GPC analysis. The remaining oligomeric mixture was emulsified in water and allowed to react with water. GPC analysis of the ethanol quenched mixture shows a distribution of fragments comprising H_12_MDI and DMPA units, a large population of unreacted H_12_MDI, and a small amount of unreacted DMPA with only a weak shoulder at the expected elution time (Figure 5a). As expected,^50^ the dominant product is the targeted H_12_MDI-DMPA-H_12_MDI trimer alongside the higher oligomers. This same pattern of molecular weight distribution is present in the PU prepolymer GPC (Figure S6). It was not possible to perform GPC on the final product as the high molecular weights of the chain extended PU polymers gives samples far too viscous to be handled when water is removed and, therefore, incompatible with appropriate solvents for GPC.

**Figure 6:**
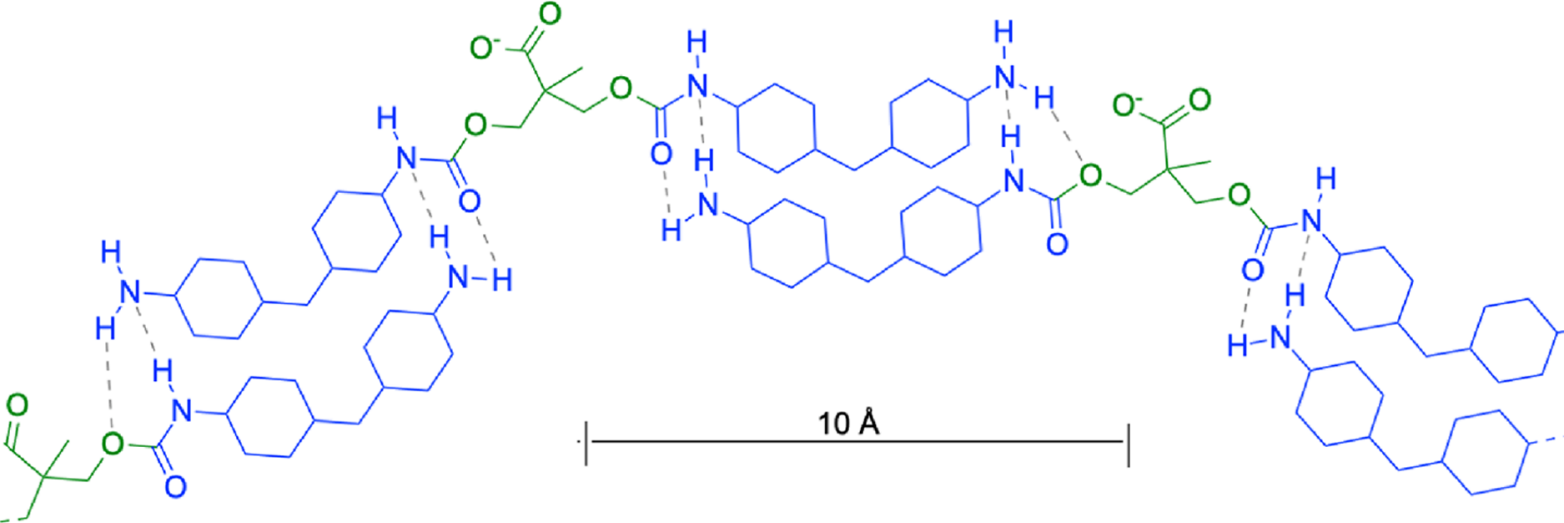
Illustration of a supramolecular structure assembly formed by hydrogen-bonded (dashed gray lines) (H_12_MDI)_2_DMPA fragments composed of hydrophobic H_12_MDI (blue) and hydrophilic deprotonated DMPA (green). The 10 Å scale bar is given as a reference.

FTIR spectrum, collected from a dried film of the H_12_MDI_DMPA dispersion, exhibits absorbances at 1700 and 1630 cm^–1^, corresponding to the expected positions of the C=O bond stretching and N–H bond bending of urethane,^59,60^ respectively (Figure 5b). However, the C=O bond signal is shifted from the expected frequency of about 1730 cm^–1^ corresponding to the free C=O bond, indicative of hydrogen bonding of urethane units in the sample.^59,60^ Similar molecules are known to form polymer-like supramolecular structures by strongly hydrogen bonding through donor and acceptor groups on neighboring molecules.^61−63^ Quantum mechanical calculations also show that urethanes form extremely strong hydrogen bonds, with energies in the region of 46–52 kJmol^–1^.^60^ Therefore, it can be expected that the collection of acidic fragments detected by GPC (Figure 5a), when taken into water, can associate to form large supramolecular structures through this hydrogen bonding (Figure 6). There is rotational freedom in the molecules, which could result in a structure mirroring that of a randomly folded polymer chain.

The SAXS profile of H_12_MDI_DMPA dispersion, diluted to 1.5 wt % in water, shows features similar to the excess scattering observed in the PUD SAXS profiles in the region of interest at *q* > 0.03 Å^–1^ (Figures 3 and 5c). This indicates that the structure formed by the H_12_MDI-DMPA molecules—namely, the folding of the supramolecular structures—is likely to be the same. The upturn of X-ray scattered intensity at *q* < 0.03 Å^–1^ indicates that larger structures, such as large aggregates, have formed in the solution of H_12_MDI_DMPA supramolecular structures that are not present in the PUD samples. This could be a result of the difference in the total charge density across the solution or the extra solvent required in the synthesis to reduce viscosity prior to sample emulsification, effecting how the structures initially form in water.

The combination of MALDI, GPC, and SAXS results of H_12_MDI_DMPA (Figure 5) strongly indicates that the excess scattering observed in PU dispersions (Figure 3) is likely related to the H_12_MDI-DMPA acidic fragments, which can form during the prepolymer reaction (Figure 4). They have amine end groups and hydrogen bond to form a supramolecular polymer-like structures (Figure 6). These fragments should form during the prepolymer emulsification in water, prior to chain extension, and, therefore, would be less likely to be present in products made by the acetone process, which chain extends prior to emulsification. As these fragments are highly acidic, the acid content of the spherical particle population is reduced compared to the original formulation, justifying the need for higher acid contents to form stable spherical particles when using the prepolymer mixing process, compared to the acetone process.^13^

In this case, the total scattering produced by the PU dispersions (eq 2) can be represented as a result of scattering from a two-population system (*m* = 2) comprising the contributions from spherical particles (eqs 5 and 6) (*i* = 1) and supramolecular structures (eqs 7–9) (*i* = 2) (Figure 3d), assuming, for the sake of simplicity, no dispersity for the parameters of the second population.

The reactants used in the synthesis of the initially targeted PU polymer composition should be redistributed among the two populations of scattering objects. If the supramolecular structures (population 2) are composed of HB components only (Figures 5 and 6), the PU composing the spherical particles (population 1) should contain less HB than the original (targeted) formulation given in Table 1. Thus, according to the law of conservation of mass, the total mass of the components must remain unchanged from the preparation of samples, and this condition must be counted in the SAXS modeling.^66^ The total volume fraction of PU in each sample (ϕ_tot_) is expressed as
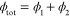
10where ϕ_1_ and ϕ_2_ are the volume fraction of spherical PU particles and hydrogen-bonded acidic fragments of the supramolecular structure, respectively. ϕ_tot_ is known as it can be calculated from the total mass of the components used for the sample preparation and the average PU mass density measured by liquid densitometry (Figure S2 and Table S2). Thus, if one of the volume fraction parameters is fitted, then the other should be calculated using the confinement imposed by eq 10. The volume fraction of population 2 affects the PU composition of the particles (population 1), and, therefore, their SLD (eq S11) and this must also be taken into account.

Since the supramolecular structure is composed of HB fragments, its SLD, ξ_2_, can be calculated before the SAXS analysis using the estimated chemical composition (Figure 6) and HB mass density measured by a liquid densitometer (Figure S3) (eq S2). However, the calculations of the spherical particle SLD, ξ_1_, taking into account the fact that the population 2 volume fraction affects the PU composition of the population 1, must be included in the model for SAXS data analysis (eq S11).

Dispersity of only one parameter (the particle radius) is considered for population 1 (eq 5), and no dispersity is assumed for the parameters of population 2. Direct methods of size distribution analysis for the primary population of PU spherical particles are not possible due to the structure factor arising from particle charge repulsion, even at low concentrations, and the presence of the population of supramolecular structures contributing to the total scattering. Instead, various distribution functions were considered for the particle radius to determine the most appropriate one. A Gaussian distribution of particle radii (eq S19) gave either satisfactory or poor fits to the experimental SAXS data, due to the presence of larger particles skewing the distribution, particularly in HB60_SS650. However, the log-normal distribution (eq S20) provided good fits to the experimental data. Such distribution is known to be common to commercial particulate systems, including those produced via emulsion polymerization, colloidal precipitation reactions, and dispersions achieved through comminution,^64,65^ so this distribution was selected for the SAXS model to describe Ψ_1_(*r*) (eq 5). Finally, it was assumed in the SAXS analysis that interactions between supramolecular structures, forming the minor population, have a weak effect on the scattering profiles, and therefore, it was taken that *S*_2_(*q*) = 1.

The developed two-population SAXS model gives good fits to the experimental scattering profiles of all PUD compositions (Figure 3 and Table 2). The particle diameter obtained for the HB50_SS650 sample, 2*R* = 35.8 nm (Table 2), is consistent with the AFM image, which suggests a diameter of approximately 40 nm (Figure 2a). The fitting results show that the size of the supramolecular structures is reasonably consistent between the samples, with *R*_g_ values in the range 2.1–2.9 nm. The excluded volume parameter of the supramolecular structures, found to be in the range of 0.31 ≤ ν ≤ 0.34 for all PUDs, is significantly below a value of 0.5 corresponding to “theta” solvent conditions.^66^ This deviation indicates that population 2 comprises structures similar to collapsed polymer chains in a “poor” solvent. This is likely due to the incompatibility of the polar aliphatic PU components that make up the supramolecular fragments and water. It can be expected that the fragments dissolved during the emulsification step of the synthesis when the prepolymer solvent, MEK, was still present in the solution. On removal of the MEK from the final dispersion, the solvent becomes less ideal, leaving collapsed supramolecular structures in the aqueous dispersions. This also explains the excessive foaming observed on removal of MEK as the acidic fragments exhibit surfactant behavior at the MEK–water interface.

**Table 2:** Parameters Obtained from the Simultaneous Fitting of an Analytical Expression of Scattering Intensity Combining Contributions from Spherical Particles and Collapsed Hydrogen-Bonded Polymer Chains^a^

	spherical particle parameters		hydrogen-bonded supramolecular structures parameters		
PUD sample	*R* /nm	σ*	ϕ_2_ /ϕ_tot_	*R*_g_ /nm	ν
H_12_MDI_DMPA				2.2	0.33
HB50_SS650	17.9	1.21	0.028	2.8	0.31
HB50_SS1000	12.0	1.35	0.105	2.1	0.31
HB50_SS2000	8.3	1.32	0.208	2.2	0.34
HB60_SS650	15.0	1.14	0.108	2.8	0.34
HB60_SS1000	10.1	1.15	0.203	2.8	0.34
HB60_SS2000	9.7	1.19	0.329	2.5	0.32
HB70_SS650	15.3	1.09	0.209	2.9	0.34
HB70_SS1000	13.0	1.14	0.283	2.6	0.34
HB70_SS2000	13.0	1.09	0.394	2.2	0.34

a *R* is the geometric mean particle radius with multiplicative standard deviation σ*. The proportion of PU distributed as supramolecular structures is quantified by ϕ_2_/ϕ_tot_. *R*_g_ gives the radius of gyration of these structures, and ν is a structural parameter that indicates solubilization of these polymer-like structures.

**Figure 7:**
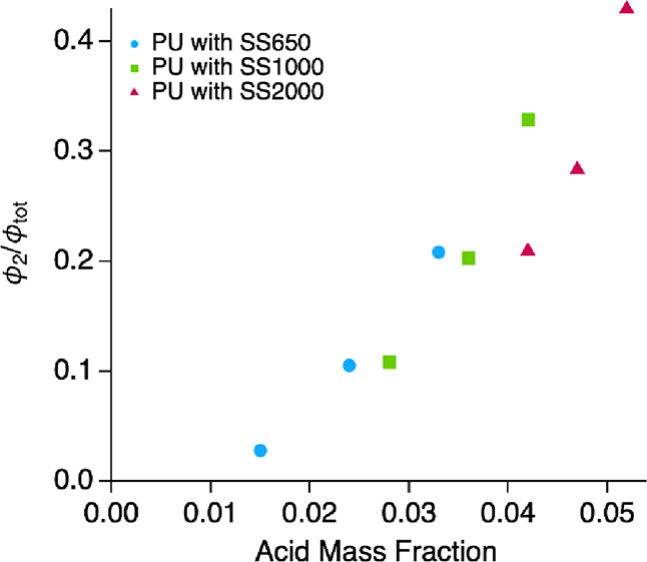
Relationship between the acid (COOH) content in the original PU formulation (Table S1) and the fraction of PU in supramolecular structures formed by (H_12_MDI)_*n*+1_DMPA_*n*_ molecules, measured by SAXS, (Table 1, ϕ_2_/ϕ_tot_) for PUD samples synthesized with polyether (soft segment) of *M*_n_ = 650 g mol^–1^ (blue circles), 1000 g mol^–1^ (green squares), and 2000 g mol^–1^ (red triangles).

The SAXS results show that the fraction of the PU distributed as acidic fragments in supramolecular structures (quantified as ϕ_2_/ϕ_tot_, Table 2) has a strong dependence on the acid content (Figure 7). As expected, the higher proportions of DMPA in the formulation increase the likelihood of forming HB-rich molecules.

This trend in the distribution of the PU between supramolecular structures of acidic fragments and spherical particles can also be confirmed in the AFM images (Figure 2). Spherical particles are clearly observed in the AFM image collected on the spin-dried sample of HB50_SS650 (Figure 2a), which contains the lowest proportion of supramolecular structures (2.8 vol % of the total PU, Table 2). The resolution of the spherical particles becomes worse as the proportion of supramolecular structures of acidic fragments increases to 10.5 and 20.8 vol % for HB50_SS1000 (Figure 2b) and HB50_SS2000 (Figure 2c), respectively, despite the decrease in particle size dispersity, σ* (Table 2). This is thought to be because during drying the supramolecular structures localize between spherical particles reducing the ability of AFM to resolve the particle interfaces. This is further supported by the reduction in root-mean-square roughness of the surface coverage formed by the PUDs with increasing SS *M*_n_: 4.9 ± 0.9 nm for 650 g mol^–1^, 1.76 ± 0.23 nm for 1000 g mol^–1^, and 0.93 ± 0.15 nm for 2000 g mol^–1^, as estimated from AFM (Figure 2), indicating that the surface becomes smoother as the concentration of supramolecular structures increases. The trend observed in the relationship between the acid content and fraction of the secondary supramolecular structures (Figure 7) suggests that to avoid the formation of the supramolecular structure during PUD synthesis via the prepolymer mixing process, the acid mass fraction in the reaction mixture should be about 0.012 or less.

**Figure 8:**
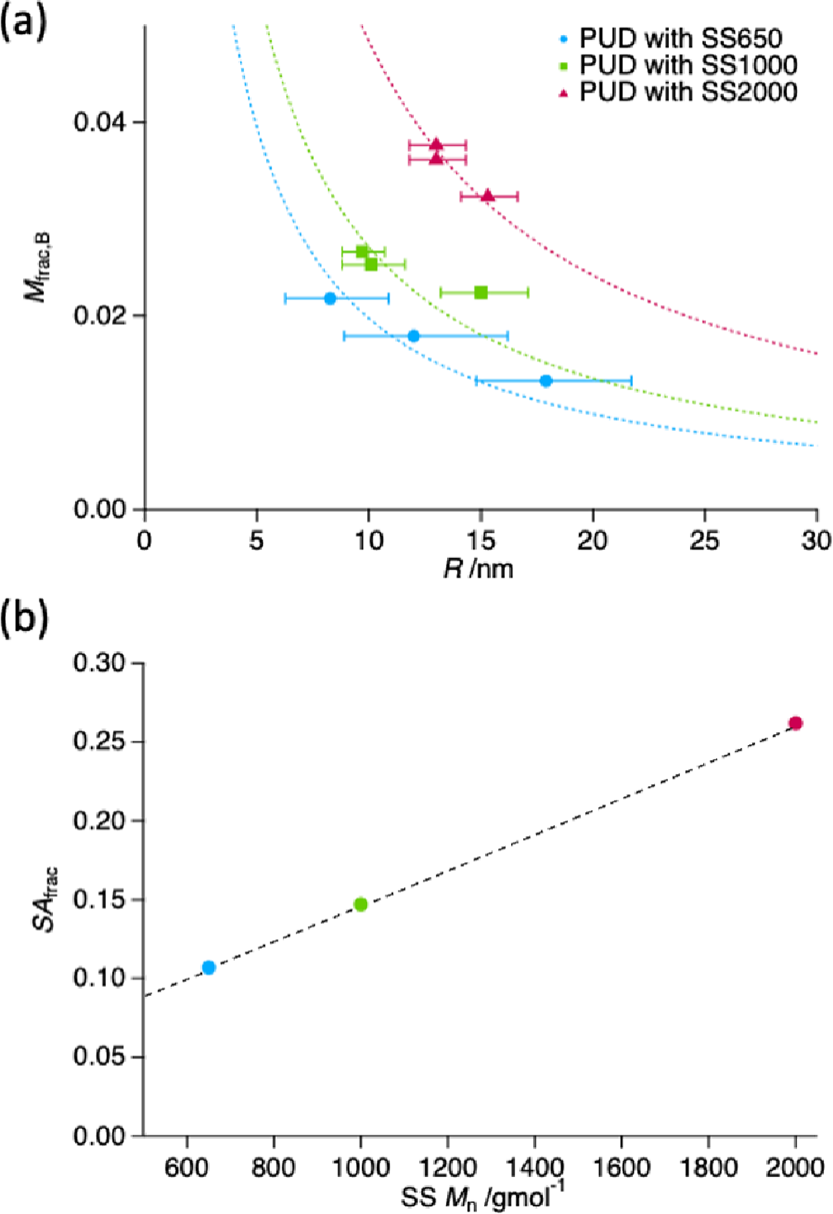
(a) Dependence of geometric mean particle radius, *R*, on the mass fraction of acid groups in the PU particles, *M*_frac,B_, calculated from the volume fraction of dissolved chains for PUD composed of PTMO with *M*_n_ = 650 g mol^–1^ (blue crosses), 1000 g mol^–1^ (green crosses), and 2000 g mol^–1^ (red crosses). Error bars indicate the multiplicative standard deviation. The dotted curves show fits of the PSC model^34^ adapted for wt % acid group content (eqs 2–7). (b) The fraction of the particle surface covered by hydrophilic B units (SA_frac_) required for stable spherical particles of PU in dispersion with respect to PTMO *M*_n_ in the soft segment of PU. A dashed straight line is given for guidance.

SAXS results obtained for population 1 show that the uniformity of the particle size improves with an increase in soft-segment molecular weight, as indicated by the decrease in σ* (Table 2). For PUDs comprising SS with the same *M*_n_, the particle radius decreases with increasing HB content, related directly to the amount of acidic groups stabilizing the PU spherical particles (Table 2). The acid content in the PU spherical particles can be calculated from SAXS results by subtracting the acid content that is found in the second population [assuming that the supramolecular structure is composed of the most abundant (H_12_MDI)_2_DMPA species, Figure S6] from the total amount used in the initial PU formulation (eqs S12–S18 and Table S3). The correlation between the particle radius and hydrophilic acid group content observed for PUDs (Figure 8a) is consistent with the PSC model.^34,35^ As there is a statistical element to the formation of PUs (the distribution of diol and diamine between diisocyanates) and the acidic group of DMPA acts as a hydrophile in an otherwise hydrophobic chain, the PSC model, initially developed for amphiphilic statistical copolymers, could be adapted for PUDs. The acidic moieties fulfill the role of the hydrophilic (anionic) B units; however, the hydrophobic A units, in the case of PUDs, are made up of PTMO, H_12_MDI, and ethylene diamine.

According to the PSC model, the number of hydrophilic B units per particle (*H*_B_) can be estimated by
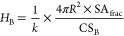
11where SA_frac_ is the fraction of the particle surface covered by B units, CS_B_ is the cross-sectional area of a single B unit, and *k* is the fraction of B units found at the particle interface. In the case of acrylic statistical copolymers, SANS measurements have shown that *k* is about 0.5, indicating that half of the hydrophiles are at the particle surface and the other half are trapped in the interior.^35^ It follows that the number of hydrophobic A units (*H*_A_) can be estimated from
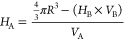
12where *V*_B_ is the B unit volume and *V*_A_ is the hydrophobic A unit volume. Knowing *H*_B_ and *H*_A_, the mole fraction of hydrophilic units (χ_B_) can be calculated:
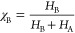
13

The concentration of hydrophile at the particle/water interface, represented by SA_frac_, is the key parameter in the control of particle size. SA_frac_ is dependent on the copolymer composition and is obtained from the experimental data fitting. Application of the PSC model to the PUDs is complicated by the fact that the A units are represented by three different hydrophobes and their ratio changes depending on the formulation. Subsequently, there is no appropriate *V*_A_ value that is independent of the composition. Due to the complexity of PU formulations, it was deemed that modifying the PSC model as an expression for calculating mass fraction (and not mole fraction) of acid groups would be more preferable. This can be done by converting *H*_B_ to the mass of B units per particle (*M*_B_) using
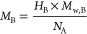
14where *M*_w,B_ is the B unit molecular weight and *N*_A_ is Avogadro’s number. The mass fraction of acidic B units in the particles (*M*_frac,B_) is given by
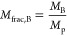
15where the mass of the entire particle (*M*_p_) can be found by
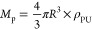
16and ρ_PU_ is the average mass density of the whole particle. Due to the minimal deviations in ρ_PU_ across the range of samples (Table S2), an average value of 1.10 g cm^–3^ was used.

For PUDs, it is expected that any acidic B unit trapped in the interior of the particle would cause an influx of water, which, in turn, would have an effect on the SLD of the particles. However, the latter has not been indicated by the SAXS analysis performed. Thus, it was assumed that *k* = 1.

The PSC model adopted for the PUDs fits the experimental data well (Figure 8a), with values in a range similar to those obtained for similarly amphiphilic statistical copolymers of acrylic. The analysis shows that particles of a given size containing a soft segment of higher *M*_n_ require a greater content of acidic units, which increases their areal density on the particle surface. It would be logical to expect that a higher molecular weight soft segment is more hydrophobic, and therefore, the SA_frac_ required for stable particles is greater (Figure 8b and Table S4). This is in good agreement with the results obtained for acrylic statistical copolymers demonstrating a linear relationship between the fraction of the nanoparticle surface covered by the hydrophilic component and the partition coefficient values of the hydrophobic comonomer.^35^

## Conclusions

A set of aqueous dispersions of PU, composed of H_12_MDI, DMPA and ethylenediamine forming hard blocks (HBs), and PTMO soft segments, with variable hard block/soft-segment mass ratio (50/50, 60/40, and 70/30), soft-segment length, and acid content, has been produced using the prepolymer mixing method. In this multistep method, isocyanate-capped prepolymer fragments were synthesized in MEK, neutralized with triethylamine, and then dispersed in water and chain extended with ethylenediamine. It is found that the synthesis of PUDs produces byproducts, namely, a water-soluble population of acidic fragments composed of *n* + 1H_12_MDI and *n*DMPA, formed during the PU prepolymer synthesis. H_12_MDI-DMPA-H_12_MDI [or (H_12_MDI)_2_DMPA] trimers were detected by MALDI and GPC as the dominant species. Upon emulsification, the isocyanate end-caps react with water to give amine groups, and these molecules strongly hydrogen bond, as indicated by FTIR, forming supramolecular structures detected by SAXS and confirmed by AFM. Thus, these PU dispersions represent a system composed of two populations: PU macromolecules, composed of HBs and SSs, self-assembled in spherical particles (main population) and water-soluble supramolecular structures formed by hydrogen-bonded H_12_MDI and DMPA acidic fragments (secondary population). As the secondary population is a consequence of the sequence of steps in the prepolymer mixing process, these findings reveal the cause of the loss in product quality compared with those made by the acetone method. Since this secondary population removes acid from the spherical particles of the main population, this justifies the need for higher acid contents to make stable spherical particles when using the prepolymer mixing method compared with the acetone method.

A SAXS analytical model was developed to measure the distribution of PU components between the charge-stabilized spherical particles and supramolecular structures. The model, based on conservation of mass, counts the total PU formulation, composition of the acidic fragments, mass density of the components, and scattering length density of the populations. Analysis of PUD scattering patterns using the model developed has shown that the proportion of acid-rich supramolecular structures increases with the DMPA content. Fitting analytical models to the experimental SAXS results has demonstrated that the intensity of scattering originating from the supramolecular structures can be described by the form factor of a Gaussian chain corresponding to polymers in solution.

PU molecules, forming the main population of spherical particles, can be represented as amphiphilic statistical copolymers. It is shown that the PSC model, originally developed for characterization of self-assembled acrylic statistical copolymers, can be adopted for characterization of the PUD particle radius. It is assumed in the model that the acidic groups of DMPA, acting as a hydrophile in an otherwise hydrophobic chain, are localized at the particle surface. It was found (in analogy to the acrylic copolymers) that the PU particle radius follows the PSC model predictions and is controlled by the DMPA (acid) content in the PU molecules, where an increase in PU acidity resulted in a decrease in particle size. The particle surface area fractional coverage of acid required for stable spheres increases with soft segment *M*_n_, from 0.107 for *M*_n_ ≈ 650 g mol^–1^ to 0.262 for *M*_n_ ≈ 2000 g mol^–1^. This also follows the findings of the PSC model that a more hydrophobic comonomer requires a greater surface area coverage of acid groups for stable spherical particles.

## Supporting Information Available

Details of formulations, synthesis, AFM, MALDI, FTIR, GPC and SAXS methodologies, time-resolved GPC of the prepolymer reaction, SAXS data plotted at absolute intensity, details of the “blob” and two-population analytical SAXS models, representative fittings of the “blob” model, solution density measurements, calculations of scattering length densities, particle size distributions, and recalculated acid contents of the primary population of spherical particles (PDF)

## Abbreviations

AFM: atomic force microscopy
DMPA: dimethylolpropionic acid
FTIR: Fourier-transform infrared spectroscopy
GPC: gel permeation chromatography
HB: hard block
H_12_MDI: hydrogenated methylene diphenyl diisocyanate
MALDI: matrix-assisted laser desorption/ionization
PSC: particle surface charge
PU: polyurethane
PUD: polyurethane dispersion
SANS: small-angle neutron scattering
SAS: small-angle scattering
SAXS: small-angle X-ray scattering
SLD: scattering length density
SS: soft segment
