# Correction to “Synthesis, Morphology, and Particle Size Control of Acidic Aqueous Polyurethane Dispersions”

**DOI:** 10.1021/acs.macromol.5c00418

**Published:** 2025-03-03

**Authors:** Ellen J. Quane, Niels Elders, Anna S. Newman, Sophia van Mourik, Neal S. J. Williams, Keimpe J. van den Berg, Anthony J. Ryan, Oleksandr O. Mykhaylyk

The originally published Table 2 is incorrect. The sample names were entered in the wrong order such that they matched up with the incorrect corresponding parameters. This mistake has been resolved in the corrected Table 2 below:

**Table 2:** Parameters Obtained from the Simultaneous Fitting of an Analytical Expression of Scattering Intensity Combining Contributions from Spherical Particles and Collapsed Hydrogen-Bonded Polymer Chains^a^

	spherical particle parameters		hydrogen-bonded supramolecular structures parameters		
PUD sample	*R* (nm)	σ*	ϕ_2_/ϕ_tot_	*R*_g_ (nm)	ν
H_12_MDI_DMPA				2.2	0.33
HB50_SS650	17.9	1.21	0.028	2.8	0.31
HB60_SS650	12.0	1.35	0.105	2.1	0.31
HB70_SS650	8.3	1.32	0.208	2.2	0.34
HB50_SS1000	15.0	1.14	0.108	2.8	0.34
HB60_SS1000	10.1	1.15	0.203	2.8	0.34
HB70_SS1000	9.7	1.19	0.329	2.5	0.32
HB50_SS2000	15.3	1.09	0.209	2.9	0.34
HB60_SS2000	13.0	1.14	0.283	2.6	0.34
HB70_SS2000	13.0	1.09	0.394	2.2	0.34

a *R* is the geometric mean particle radius with multiplicative standard deviation σ*. The proportion of PU distributed as supramolecular structures is quantified by ϕ_2_/ϕ_tot_. *R*_g_ gives the radius of gyration of these structures, and ν is a structural parameter that indicates solubilization of these polymer-like structures.

The caption to the originally published Figure 7 refers to the tables incorrectly. The first reference should be made to Table 1 and not to Table S1. The second reference should be made to Table 2 and not to Table 1. The value ϕ_2_/ϕ_tot_ is given in Table 2, as specified in the corrected caption below:

Corrected Figure 7 caption: Relationship between the acid (COOH) content in the original PU formulation (Table 1) and the fraction of PU in supramolecular structures formed by (H_12_MDI)_*n*+1_DMPA_*n*_ molecules, measured by SAXS (Table 2, ϕ_2_/ϕ_tot_) for PUD samples synthesized with polyether (soft segment) of *M*_n_ = 650 g mol^–1^ (blue circles), 1000 g mol^–1^ (green squares), and 2000 g mol^–1^ (red triangles).

