# Supplementary material for: Synthesis, Morphology, and Particle Size Control of Acidic Aqueous Polyurethane Dispersions
Source: Macromolecules. 2024 Nov 14;57(22):10623–34. doi: 10.1021/acs.macromol.4c02046 (PMC11603786; doi:10.1021/acs.macromol.4c02046)
Supplement: Supplementary file 1 — ma4c02046_si_001.pdf [file ma4c02046_si_001.pdf]

Supporting Information

Synthesis, Morphology, and Particle Size Control of Acidic Aqueous Polyurethane Dispersions

Ellen J. Quane,[§ †] Niels Elders,[‡] Anna S. Newman,[§] Sophia van Mourik,[§] Neal S. J. Williams,[$] Keimpe J. van den Berg,[‡] Anthony J. Ryan[§] and Oleksandr O. Mykhaylyk[§]*

§ School of Mathematical and Physical Sciences, The University of Sheffield, Dainton Building, Brook Hill, Sheffield, South Yorkshire, S3 7HF, UK.

† Centre for Analysis and Synthesis, Lund University, Naturevetarvägen 22, 223 62, Lund, SE.

‡ Akzo Nobel Car Refinishes BV, Department of Resin Technology, Rijksstraatweg 31, 2171 AJ Sassenheim, NL.

$ CPI, The Coxon Building, John Walker Road, NETPark, Sedgefield, County Durham, TS21 3FE, UK.

Contents

Synthesis 2

Synthesis of polyurethane dispersions 2

Synthesis of H_{12}MDI-DMPA supramolecular structures 4

Atomic Force Microscopy (AFM) 4

Small angle X-ray scattering (SAXS) analysis 5

Data collection 5

SAXS data plotted on absolute intensity scale 5

Calculation of scattering length density (SLD) of studied materials 6

Scattering form factor of a spherical PU particle with water pockets 8

Two-population SAXS Model 9

Gaussian distribution of particle radius 12

Lognormal distribution of particle radius 12

Matrix Assisted Laser Desorption Ionisation (MALDI) Time of Flight Mass Spectrometry 13

Gel permeation chromatography (GPC) 14

Fourier Transform Infrared Spectroscopy FTIR 16

PSC Model Fitting 16

References 17

Synthesis

Synthesis of polyurethane dispersions

Polyurethane dispersions (PUDs) were synthesised using hydrogenated methylene diphenyl diisocyanate (H_{12}MDI), dimethylolpropionic acid (DMPA), and ethylenediamine forming hard blocks (HBs), and poly(tetramethylene oxide) (PTMO) composing soft segments (SSs) of the PU

molecules (Table S1). Samples were produced using three molecular weights of PTMO, at three HB contents.

Table S1. Details of synthetic steps and material formulations used in the synthesis of PUD samples.

Synthetic step	Component	Samples								
		HB50_ SS650	HB50_ SS1000	HB50_ SS2000	HB60_ SS650	HB60_ SS1000	HB60_ SS2000	HB70_ SS650	HB70_ SS1000	HB70_ SS2000
PU pre-polymer synthesis	PTMO M_n= 650 gmol^{-1} (g)	95.90	-	-	75.56	-	-	56.68	-	-
	PTMO M_n=1000 gmol^{-1} (g)	-	95.74	-	-	75.18	-	-	56.19	-
	PTMO M_n=2000 gmol^{-1} (g)	-	-	95.01	-	-	74.86	-	-	56.22
	Dimethylol propionic acid (g)	8.70	13.70	18.47	15.97	19.99	23.66	23.26	26.36	29.06
	Hydrogenated methylene diisocyanate (g)	83.60	77.87	72.9	92.62	88.26	84.18	102.59	99.45	96.37
	Methyl ethylketone (g)	47.06	46.82	46.60	46.92	46.73	46.55	46.51	46.37	46.28
	Dibutyl tin dilaurate (g)	0.10	0.10	0.10	0.10	0.10	0.10	0.10	0.10	0.10
	Triethylamine (g)	0.13	0.21	0.28	0.24	0.30	0.36	0.35	0.40	0.44
Dilution	Methyl ethylketone (g)	78.41	78.05	77.65	75.85	75.56	75.25	75.18	78.50	74.82
Neutralisation	Triethylamine (g)	6.10	9.60	12.94	11.20	14.01	16.59	16.31	18.48	20.37
Emulsification	Water (g)	464	470	475	468	472	477	478	494	484
Chain extension	Ethylenediamine (g)	4.47	4.17	3.90	4.95	4.73	4.86	5.50	5.31	5.17
	Water (g)	17.88	16.68	15.60	19.8	18.92	19.44	22.00	21.24	20.68
MEK evaporation	Methyl ethylketone (g)	-125.47	-124.87	-124.25	-122.77	-122.29	-121.80	-121.69	-124.87	-121.1
Final PUD solids content (wt. %)		30.7	32.9	26.9	24.7	25.0	23.3	18.2	19.7	16.9

All PU prepolymers were designed at an NCO/OH molar ratio of 1.5 and made following the prepolymer mixing method. A 1 L 4-necked flask (under N_2 atmosphere), equipped with a mechanical stirrer, a reflux condenser, and a thermocouple, was charged with PTMO, DMPA and H_{12}MDI. Methyl ethylketone (MEK) was added to dilute the PU monomer constituents to 80 wt.%. Dibutyl tin dilaurate (DBTDL) (0.05 wt.% with respect to the mass of PU monomers) was added as catalyst, and triethylamine (TEA) (2 mol.% relative to DMPA content) was added to increase the solubility of DMPA by deprotonation of a portion of the acid groups. The reaction mixture was heated to 95 °C while applying mild agitation. After 4 hours reaction time, the PU prepolymer solution was cooled to 50 °C while simultaneously diluting further to 60 wt% PU by slowly adding MEK. Further TEA (93 mol% with respect to DMPA content) was added allowing 5 minutes for neutralisation of the carboxylic acid groups to occur. Next, the neutralised PU pre-polymer solution in MEK, at 50 °C, was added to a 1 L tube reactor containing water at 30 °C (PU solution / water ratio 40/60) over 3 minutes while stirring well. The emulsified PU prepolymer was chain extended

by addition of aqueous ethylene diamine solution (20 wt% in water) over 10 minutes. Finally, MEK was removed from the PUD by vacuum distillation (<80 mbar at 50 °C) and the dispersion filtered through a 125 µm gauze.

Synthesis of H_{12}MDI-DMPA supramolecular structures

A 50 mL round bottom flask (under N_2 atmosphere), equipped with a reflux condenser, was charged with DMPA (1.28 g, 9.55 mmol), H_{12}MDI (5.00 g, 19.1 mmol), MEK (25.11 g), DBDTL (0.01 g) and TEA (0.96 g, 9.55 mmol). The resulting solution was heated for 4 hours at 65 °C while stirring. Full conversion was measured using isocyanate back titration, and a 0.5 g sample for GPC analysis taken at this point (quenched in 5 g ethanol + one drop DBTDL). Next, the temperature of the 20 wt% PU-prepolymer solution in MEK was lowered to 50 °C and the solution transferred to a flask containing 250 mL of water at 30 °C, with stirring. The resulting dispersion was stirred for 24 hours at 40 °C to enable the NCO/water reaction. Finally, MEK was removed from the PUD by vacuum distillation (<60 mbar at 50 °C) using a rotary evaporator.

Atomic Force Microscopy (AFM)

AFM samples were prepared by spin coating each PUD onto single-crystal silicon wafer at 5000 rpm for 30 seconds. To preserve the morphology of PU particles in dispersion, AFM samples were prepared from low-concentration dispersions to ensure rapid drying. Thus, prior to the spin coating, the as-synthesised PUDs (Table S1) were diluted to by a factor of ten using deionised water. The silicon wafer surfaces were preliminarily cleaned for 5 minutes using a Henniker oxygen plasma oven.

AFM imaging of the PUs was performed at ambient conditions using tapping mode on a Dimension 3100 scanning probe microscope (Veeco, New York, USA) equipped with a Nanoscope 3A feedback controller. SCOUT 350 RAI probe tips (NuNano, Bristol, UK) were used with a resonance frequency near 340 kHz. All AFM images were processed using the open-source software Gwyddion (version 2.59).[1]

Small angle X-ray scattering (SAXS) analysis

Data collection

SAXS patterns were recorded using a Xeuss 2.0 laboratory beamline (Xenocs, Grenoble, France), equipped with a liquid gallium Metal-jet X-ray source (Excillum, Kista, Sweden) (wavelength of X-ray radiation λ = 1.34 Å) and a Pilatus 1M two-dimensional hybrid pixel detector (Dectris, Barden-Daetwil, Switzerland). Scattering data were collected over the range of 0.007 $Å^{-1} \leq q \leq 0.2$ $Å^{-1}$. The length of the scattering vector, q, is defined as

$$q = \frac{4\pi \sin\theta}{\lambda} \qquad \text{(S1)}$$

where θ is half the scattering angle. The measurements were performed at a temperature of 20 °C. Two-dimensional scattering patterns were integrated to give one-dimensional profiles and calibrated to absolute intensity (scattering of pure water was used as a reference standard) using the XSACT software package supplied with the instrument. Further SAXS data processing (background subtraction) and analysis (SAXS model fitting to scattering profiles) were carried out using Irena SAS macros for Igor Pro software.[2]

SAXS data plotted on absolute intensity scale

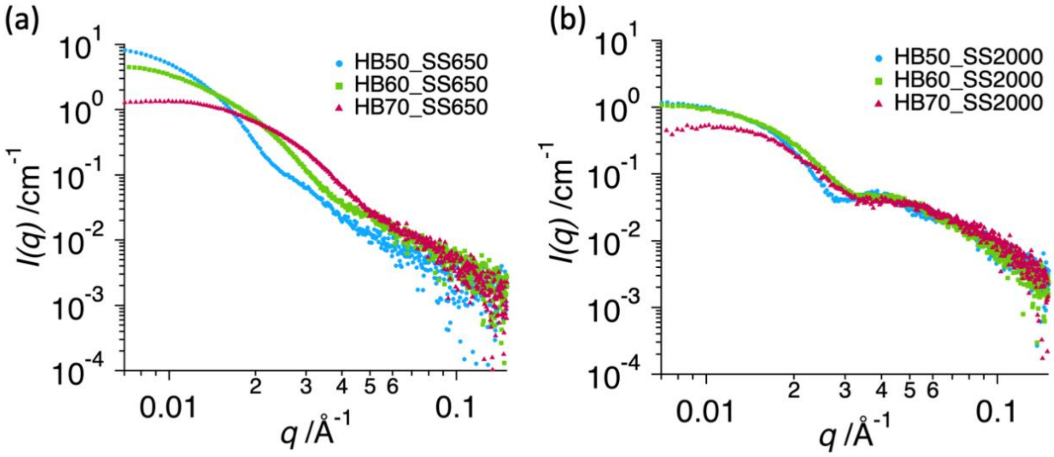

Figure S1. SAXS profiles for 1 wt.% aqueous dispersions of PU, composed of SS of M_n: a) 650 $gmol^{-1}$ and b) 2000 $gmol^{-1}$, with HB content of 50 wt.% (blue circles), 60 wt.% (green squares), and 70 wt.% (red triangles).

Calculation of scattering length density (SLD) of studied materials

The SLD of compound C (ξ_{C}) is given by:

$$\xi_{\mathrm{C}} = \frac{b_{\mathrm{e}}\rho_{\mathrm{C}}N_{\mathrm{A}}}{M_{\mathrm{C}}}\sum_{i=1}^{K} n_i z_i \qquad \text{(S2)}$$

where b_{e} is the scattering length of one electron, ρ_{C} is the mass density of compound C, N_{A} is Avogadro's number, M_{C} is the molecular mass of the compound C (or one repeat unit of polymer), K is the number of atom types in the compound, n_i is the number of ith *type* atoms in the compound, and z_i is the number of electrons in the ith type atom.

In order to find ρ_{C} for each PU polymer, mass densities of the aqueous PUDs (ρ_{PUD}) were measured using an Anton Paar DMA 5000M U-tube style liquid densitometer. All measurements were performed at 20 °C. The equipment was calibrated using deionised water assuming its mass density at 20 °C as 0.9982 gcm^{-3}.[3] Measurements were carried out at four PU concentrations, and for pure water. The PU polymer density (ρ_{PU}) (Table S2) was found by fitting:

$$\rho_{\mathrm{PUD}} = \frac{1}{\frac{1 - C_{\mathrm{tot}}}{\rho_w} + \frac{C_{\mathrm{tot}}}{\rho_{\mathrm{PU}}}} \qquad \text{(S3)}$$

where ρ_{PUD} and C_{tot} are the measured density of the PU dispersion and mass concentration of PU in the dispersion, respectively, and ρ_{w} is the mass density of water (Figure S2).

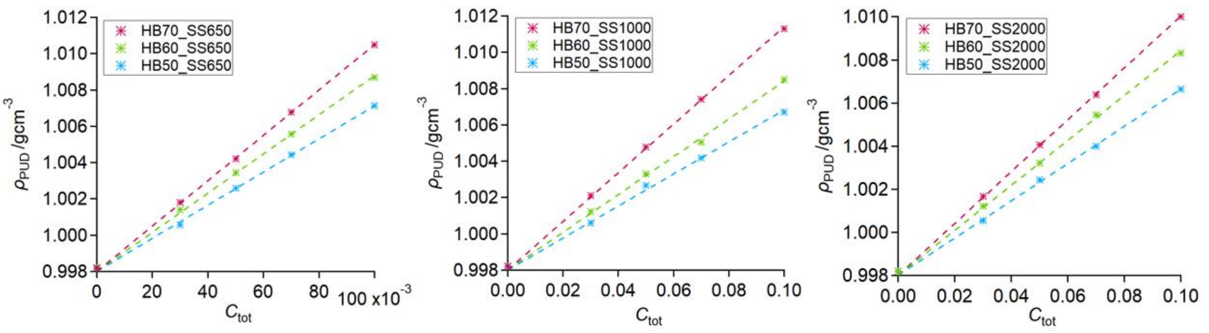

Figure S2. Solution mass density of PUD samples containing PTMO of M_n: a) 650 gmol^{-1}, b) 1000 gmol^{-1}, and c) 2000 gmol^{-1} with 50% hard block (blue crosses), 60% hard block (green crosses) and 70% hard block (red crosses). The dashed lines show the fit of Equation S8 to each data set used for the mass density calculations of PU polymers.

Table S2. Average mass densities (ρ_{PU}) and scattering length density (ξ_{PU}) of PU polymers in aqueous PUDs calculated from mass density measurements (Figure S2), and average scattering length density of PU spherical particles, population 1, (ξ_1) obtained from SAXS analysis.

Sample	ρ_{PU} /gcm^{-3}	ξ_{PU} /10^{10} cm^{-2}	ξ_1 /10^{10} cm^{-2}
HB50_SS650	1.0932	10.10	10.07
HB60_SS650	1.1131	10.25	10.16
HB70_SS650	1.1344	10.41	10.14
HB50_SS1000	1.0896	10.06	9.94
HB60_SS1000	1.1085	10.20	10.11
HB70_SS1000	1.1460	10.52	10.27
HB50_SS2000	1.0875	10.05	9.80
HB60_SS2000	1.1089	10.21	9.88
HB70_SS2000	1.1286	10.36	9.92

The PU series with the longest PTMO soft segment of M_n = 2000 gmol^{-1} (Table S2) was used to calculate the average mass density of hard blocks (ρ_{HB}) and soft segments (ρ_{SS}) (Figure S3), which were found to be 1.1905 gcm^{-3} and 0.9850 gcm^{-3}, respectively.

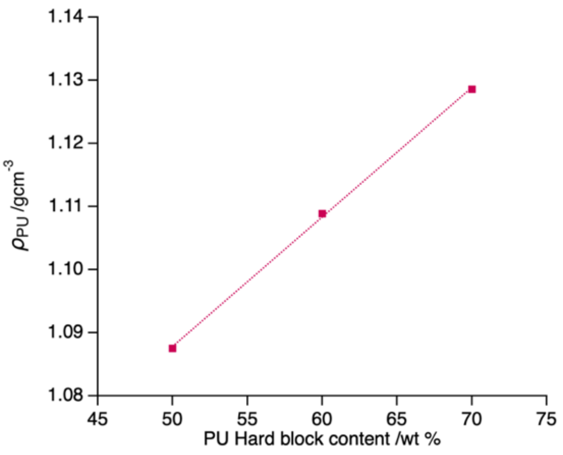

Figure S3. Mass density of PU containing PTMO soft segment of M_n = 2000gmol^{-1} (Table S2). The dotted line shows a linear fit to the data to calculate average mass density values for hard blocks and soft segments present in the synthesised PU molecules.

Scattering form factor of a spherical PU particle with water pockets

A “blob” model was proposed to describe the concentration fluctuation in micelles resulting from solvent swelling.[4] This model could be adapted to describe PU particles in dispersion with internal water-rich pockets (“blobs”) assuming that the water molecules are associated with the acidic units of the PU molecules and follow the polymer movement in the sample. Thus, applying the polymer (Gaussian coil) form factor for describing scattering originating from the water pockets, the scattering form factor of a spherical particle (Equation 6) can be modified for such PU particles with internal water as:

$$P_1(q,r) = (\Delta\xi_1)^2\big[V_1{}^2(r)A_s{}^2(q,r) - 2n_{bl}V_{bl}(R_{g,bl})\,A_{bl}\big(q,R_{g,bl}\big)A_s{}^2(q,r) + n_{bl}(n_{bl}-1)V_{bl}(R_{g,bl})^2A_{bl}{}^2\big(q,R_{g,bl}\big) + n_{bl}V_{bl}(R_{g,bl})^2D_{bl}\big(q,R_{g,bl}\big)\big] \quad \text{(S4)}$$

where $A_s(q, r)$ and $A_{bl}\ (q, R_{g,bl})$ are the normalised scattering amplitudes of a sphere, and a Gaussian coil representing the “blobs” (water pockets), respectively:

$$A_s(q,r) = \frac{3[\sin(qr) - qr\cos(qr)]}{(qr)^3} \quad \text{(S5)}$$

$$A_{bl}\big(q,R_{g,bl}\big) = \frac{1 - e^{-qR_{g,bl}}}{qR_{g,bl}} \quad \text{(S6)}$$

The form factor of a Gaussian polymer coil in Equation S4 is given by the Debye function:

$$D_{bl}\big(q,R_{g,bl}\big) = \frac{2(e^{-qR_{g,bl}} + qR_{g,bl} - 1)}{(qR_{g,bl})^2} \quad \text{(S7)}$$

$R_{g,bl}$ and $V_{bl}(R_{g,bl}) = \frac{4}{3}\pi R_{g,bl}{}^3$ are the radius of gyration and the volume of a blob, respectively, and n_{bl} is the relative number of blobs. $V_1(r) = \frac{4}{3}\pi r^3$ and r are the volume and radius of a spherical particle, respectively. $\Delta\xi_1$ is the scattering length density contrast between the PU and the water.

Substituting $P_1(q, r)$, expressed by Equation S4, into Equation 5, the intensity of scattering of the PU particles with water pockets can be obtained and used for SAXS data fitting (Figure S4).

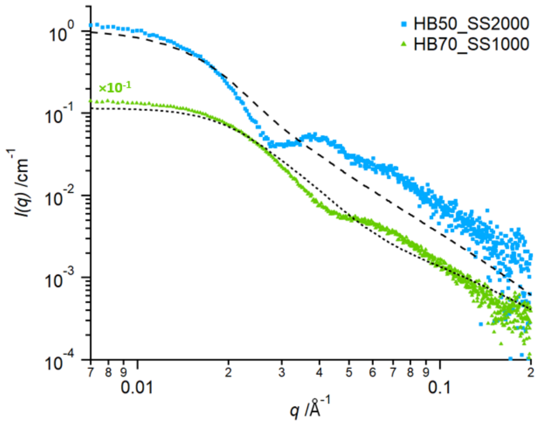

Figure S4. Best fits of the PU particles with water pockets model (Equations S6, S9-S12) (curves) to experimental SAXS profiles (symbols) of representative 1 wt.% aqueous dispersions of PU composed of soft segments of M_n = 2000 gmol^{-1} and hard block content of 50 wt.% (blue squares and dashed curve), and soft segments of M_n = 1000 gmol^{-1} and hard block content of 70 wt.% (green triangles and dotted line). To avoid SAXS data overlap, one of the profiles is offset by a factor of 0.1 (indicated on the plot).

Two-population SAXS Model

Using the general equation for scattering intensity from a multipopulational system (Equation 2), the total scattering from the synthesised PUDs can be interpreted as a signal produced by a two-population system (m = 2) composed of spherical particles (Equations 5 and 6) (i = 1) and the supramolecular structure (Equations 7- 9) (i = 2) (Figures 3d and 5). Although it is possible that there is some correlation between locations of the supramolecular structures and the spherical particles in the PUDs (e.g., supramolecular structures attach to the spherical particle surfaces), there is no evidence to suggest that this is the case. Therefore, it is assumed that they coexist as separate populations and are treated as such in the SAXS analysis.

The molecule volume, V_2, for the form factor calculation of population 2 (Equation 7) can be obtained from:

$$V_2 = \frac{M_{\mathrm{w},2}}{\rho_{\mathrm{HB}}} \tag{S8}$$

where ρ_{HB} (see Figure S3 and associated text) is used due to similarity in compositions of the acidic fragments and the hard blocks (Figure 4) and is the supramolecular structure mass density. Assuming that the supramolecular structure consists of a polymer-like single chain, $M_{w,2}$ can be calculated using molecular masses and estimated lengths of constituent monomers (10 Å and 7 Å for H_{12}MDI and DMPA, respectively), and the length of the polymer chain, L. The latter can be calculated from:

$$L = N_b b = \left(\frac{R_g\sqrt{6}}{b^{(1-\nu)}}\right)^{\frac{1}{\nu}} \tag{S9}$$

where N_b is the number of Kuhn lengths, b, in the fully extended chain, R_g is the radius of gyration of the folded polymer chain (Equation 9), and v is the excluded volume parameter related to the solvent-polymer interactions. R_g and v are obtained from SAXS data fitting, and the value of b was estimated to be 10 Å, which is approximately equal to half the length of the H_{12}MDI-DMPA-H_{12}MDI fragment (Figure 6), and is in agreement with values found in the literature for PU.[5-8] It is also assumed for the two-population model that there are negligible interactions between the supramolecular structures and, therefore, the structure factor of population 2 in Equation 2 was taken as equal to unity, $S_2(q) = 1$.

In addition, it has to be noted that the population volume fractions are constrained by the total mass of PU components used for the sample preparation and distributed between the two populations. In this respect, the total PU volume fraction, ϕ_{tot}, can be calculated from the total mass of the PU components used for the sample preparation and the average PU mass density (Figure S2 and Table S2). Thus, if one of the population volume fractions is used as a fitted parameter during SAXS analysis, the other should be calculated using the confinement imposed by Equation 10. Since the supramolecular structures (population 2) are composed of HB fragments only, its SLD, ξ_2, can be calculated (Equation S2) using the estimated chemical composition (Figure 6) and ρ_{HB} measured by liquid densitometry (Figure S3). However, the PU composition of population 1 depends on the amount of HB fragments consumed by the supramolecular structure formation. In this respect it would be more convenient to fit ϕ_2 and include in the SAXS model calculations of ϕ_1 and ξ_1 counting the ϕ_2 fitting result:

$$\phi_1 = \phi_{\text{tot}} - \phi_2 \tag{S10}$$

$$\xi_1 = \frac{\xi_{\text{PU}} - \frac{\phi_2}{\phi_{\text{tot}}} \cdot \xi_2}{1 - \frac{\phi_2}{\phi_{\text{tot}}}} \tag{S11}$$

where ξ_{PU} is the average SLD for each PU, provided in Table S2.

SAXS data analysis using this two-population model enabled the distribution of PU components between the PU spherical particle population and the supramolecular structure population to be

measured. In order to calculate the acid content of the particles comprising population 1, it was necessary to calculate the total mass fraction of PU polymers in each PUD (C_{tot}), given by:

$$C_{\mathrm{tot}} = \phi_{\mathrm{tot}} \cdot \rho_{\mathrm{PU}} \tag{S12}$$

and the mass fractions of population 1 (C_1) and population 2 (C_2):

$$C_2 = \phi_2 \cdot \rho_{\mathrm{HB}} \tag{S13}$$

$$C_1 = C_{\mathrm{tot}} - C_2 \tag{S14}$$

using ρ_{HB} due to the similarity in composition between the fragments making up the supramolecular structures in population 2 (Figure 6) and the hard block of the PU polymer (Figure 1).

The mass fraction of the PUD comprising acid groups found within population 2 is calculated from:

$$C_{\mathrm{B,2}} = C_2 \cdot \frac{M_{\mathrm{w,B}}}{M_{\mathrm{w,2}}} \tag{S15}$$

where $M_{\mathrm{w,B}}$ is the molecular weight of the acid moiety – COOH – and $M_{w,2}$ is the molecular weight of the fragments making up the second population, assuming units fragments comprising 2 H_{12}MDI and 1 DMPA as this is the most abundant species in the GPC trace (Figure S6).

The total mass fraction of acidic moieties in the PUD solution ($C_{\mathrm{B,tot}}$) is expressed as:

$$C_{\mathrm{B,tot}} = C_{\mathrm{tot}} \cdot M_{\mathrm{frac,DMPA,tot}} \cdot \frac{M_{\mathrm{w,B}}}{M_{\mathrm{w,DMPA}}} \tag{S16}$$

where $M_{\mathrm{frac,DMPA,tot}}$ is the mass fraction of DMPA in the PU polymer formulation known from synthesis (Table S1), $M_{\mathrm{w,B}}$ is the molecular weight of the acidic moiety, and $M_{\mathrm{w,DMPA}}$ is the molecular weight of a DMPA molecule.

The mass fraction of the PUD comprising acid groups found within the particles of population 1 ($C_{\mathrm{B,1}}$) is then given by:

$$C_{\mathrm{B,1}} = C_{\mathrm{B,tot}} - C_{\mathrm{B,2}} \tag{S17}$$

From this, the mass fraction of acid moieties in the particles ($M_{\mathrm{frac,B}}$) for each PU (Table S3) is expressed as:

$$M_{\mathrm{frac,B}} = \frac{C_{\mathrm{B,1}}}{C_1} \tag{S18}$$

Table S3. The mass fraction of acid groups found in spherical particles of PU in dispersion, compared to mass fraction in the entire formulation including the hydrogen-bonded supramolecular structure.

Sample	Mass fraction of acid groups	
	In total PU formulation	In Spherical particles
HB50_PE650	0.015	0.013
HB60_PE650	0.028	0.022
HB70_PE650	0.041	0.032
HB50_PE1000	0.024	0.018
HB60_PE1000	0.036	0.025
HB70_PE1000	0.047	0.036
HB50_PE2000	0.033	0.022
HB60_PE2000	0.042	0.027
HB70_PE2000	0.052	0.038

Gaussian distribution of particle radius

The probability function of a single parameter r, expressed as a Gaussian distribution, is defined as:

$$\Psi(r) = \frac{1}{\sqrt{2\pi\sigma_R^2}} e^{-\frac{(r-R)^2}{2\sigma_R^2}} \quad \text{(S19)}$$

where R is the arithmetic mean of r, and σ_R is its standard deviation.

Lognormal distribution of particle radius

The probability function of PUD particle radii, r, according to the lognormal distribution is expressed as:

$$\Psi(r) = \frac{e^{-[\ln(r)]^2/2\sigma^2}}{r\sigma\sqrt{2\pi}} \quad \text{(S20)}$$

where σ is the standard deviation of the distribution of $\ln(r)$.[9, 10]

The geometric mean radius of r, R, according to the lognormal distribution, is defined as:

$$R = e^{\bar{R}} \quad \text{(S21)}$$

where $\bar{R}$ is the arithmetic mean of the distribution of $\ln(r)$.

Similarly, the multiplicative standard deviation of R, σ^*, is related to σ by[11]

$$\sigma^* = e^{\sigma} \quad \text{(S22)}$$

By definition of a lognormal distribution, $\ln(r)$ has a normal distribution, therefore values of $\bar{R}$ and σ were found by using the fitting of a gaussian curve (Equation S19) to the distribution of $\ln(r)$ and substituted into Equations S20 and S21.

Matrix Assisted Laser Desorption Ionisation (MALDI) Time of Flight Mass Spectrometry

As-synthesised PUD samples (Table S1) were diluted with methanol (1:10) and 1.0 µl of each fraction was spotted onto a 96-well stainless steel MALDI target plate. Spots were left to dry at room temperature and then overlaid, using a Fisnar F200N automatic dispenser (flow rate of 1.2 ml hr^{-1}), with a matrix of α-Cyano-4-hydroxycinnamic acid (5 $mgml^{-1}$) dissolved in a solution of methanol containing 0.1% (v/v) trifluoracetic acid.

The data were acquired for 120 seconds over a mass range of 50 – 3000 $gmol^{-1}$ using a MALDI Synapt G2 instrument (Waters, Milford, USA) equipped with a solid-state laser with a repetition rate of 1000 Hz. The instrument was set in negative mode. For all spectra, the raw files were processed in RStudio.2023.12 and R4.3.1 integrated development environments[12] using several steps for data reduction (Table S4). The ion peak of interest [M-H]- at 604 *m*/*z* – for the $(H_{12}MDI)_2DMPA$ trimer with deprotonated acid group and additional deprotonation from the MALDI methodology - was detected along with its isotope peaks for all measured samples (Figure S5).

Table S4. Processing methods applied to MALDI spectra collected on PUDs.

Data processing step	Method
Transform intensity	Square root
Smooth data	Savitzky-Golay
Baseline correction	SNIP, iteration = 250
Normalise	Total ion count
Peak alignment	halfWindowSize = 5<br>Signal-to-noise = 4<br>tolerance = 100e-6

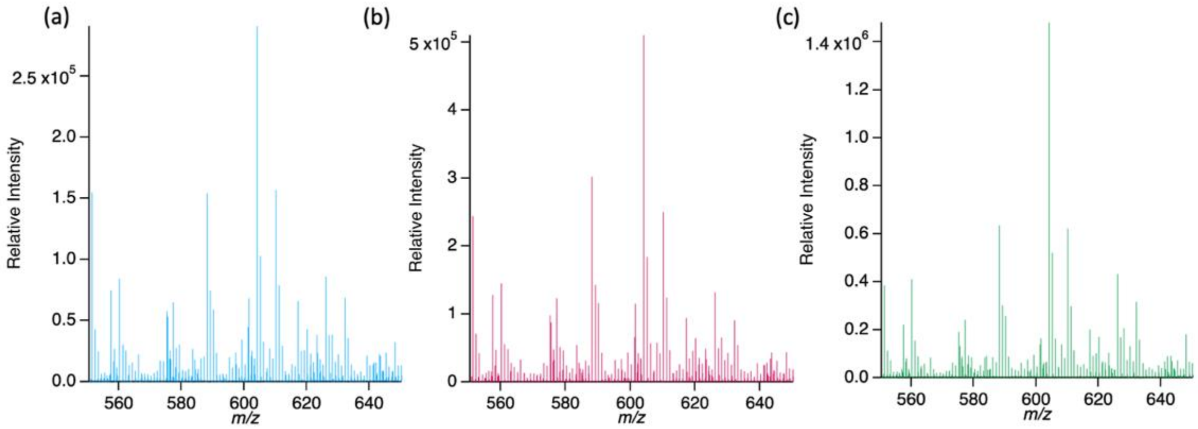

Figure S5. MALDI time of flight spectra of PUDs containing PTMO of M_n = 2000 g mol^{-1} and HB content of: a) 50 wt.% (blue), b) 60 wt.% (red), and c) 70 wt.% (green). The spectra demonstrate a pronounced peak at *m/z* = 604, indicative of deprotonated H_{12}MDI-DMPA-H_{12}MDI molecules capped with amine (product of the isocyanate reaction with water).

Gel permeation chromatography (GPC)

GPC analysis was carried out selectively on the final product of the reaction of 2:1 H_{12}MDI:DMPA (named H_{12}MDI_DMPA, Table 1), pure H_{12}MDI, pure DMPA (Figure 5a), as well as on sample aliquots of the HB60_SS1000 prepolymer reaction taken during synthesis to check the PU-prepolymer molecular weight distribution as a function of reaction time (Figure S5). For the latter, a 1 litre 4-necked flask (under N_2 atmosphere), equipped with a mechanical stirrer, a reflux condenser, and a thermocouple was charged with PTMO (M_n = 1000 gmol^{-1}), DMPA and MEK. The mixture was heated to 70 °C using an oil bath while applying mild agitation. Next, H_{12}MDI was added as fast as possible making sure the exotherm did not exceed 92 °C. The batch temperature was maintained at 92 °C and sample aliquots (~2 g) were taken after 1, 2 and 3 hours from the moment of complete H_{12}MDI addition. The free isocyanate present in the sample aliquots was quenched by diluting in 18 g dry ethanol (> 99.8 vol.%), and one drop of DBTDL added to catalyse the OH-NCO reaction at room temperature. The GPC data were collected at a flow rate of 1 ml min^{-1} using tetrahydrofuran/acetic acid as eluent, on an APC low 200 125 45 column (Waters, Milford, USA) equipped with a refractive index detector. GPC instrument was calibrated against a set of eleven low-dispersity polystyrene standards (Agilent, UK) with peak molecular weight values ranging from 580 gmol^{-1} to 72450 gmol^{-1}.

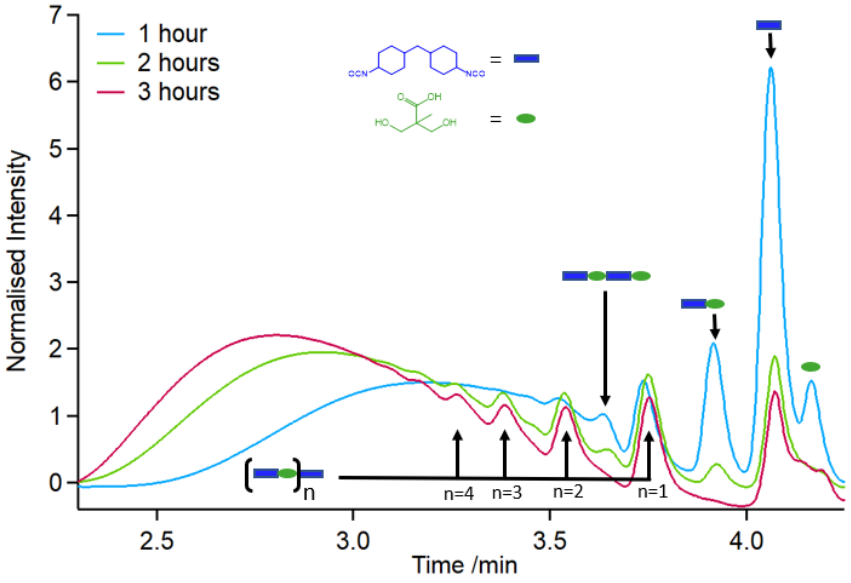

Figure S6. GPC retention time curves of sample aliquots taken at 1 hour (blue curve), 2 hours (green curve) and 3 hours (red curve) into the prepolymer reaction for the HB60_SS1000 PUD. The signal intensity is normalised by the total area under the curve within the time interval of the measurements. The prominent peaks are assigned by corresponding molecular composition including $H_{12}MDI$ (blue rectangles) and DMPA (green circles).

GPC results (Figure S6) demonstrate that the PU molecular weight distribution is changing over the time of the prepolymer rection. The GPC indicates two regions in the molecular weight (M_W) distribution. The broad peak in the short retention time region corresponding to high M_W species should originate from the PTMO-rich PU pre-polymer chains which increase in M_W while the conversion progresses. The GPC data indicate an increase in both the fraction of these molecules and their averaged M_W (shifts to shorter retention times) in the reaction mixture throughout the reaction time. A set of peaks observed in the long retention time region likely corresponds to oligomers composed of $H_{12}MDI$ and/or DMPA (Figure S6). The changes in the peak intensities indicate that the oligomer concentration reduces throughout the reaction time. As the conversion progresses, some of these oligomers react with each other to form higher M_W chains, and as such populate the higher M_W distribution. The total intensity area of the peaks originating from the oligomers in the GPC curves recorded for the sample aliquot after 1 hour of the reaction time is more than the intensity area for the sample aliquot after 3 hours. Some of the peaks (e.g., peaks at ~3.6 min and ~3.9 min) reduce in intensity to the baseline upon complete conversion. Most likely these signals originate from molecules with an equal number of $H_{12}MDI$ and DMPA (Figure S6).

Based on the observation that keeping the reaction mixture for 3 hours at 92 °C in the absence of catalyst is sufficient to reduce the intensity of these signals to the baseline, it is concluded that full conversion (no hydroxyl end groups) is reached for all PU compositions (listed in Table 1) in 4 hours of reaction time with catalyst. The peaks that remain in the low M_W region (e.g., 3.40 minutes, 3.55 minutes and 3.75 minutes) most likely originate from isocyanate-capped molecules composed of *n*+1 H_{12}MDI and *n* DMPA (Figure S6).

Fourier Transform Infrared Spectroscopy FTIR

Fourier Transform Infrared (FTIR) spectra of PU samples were recorded on a Spectrum RX FTIR Spectrometer (PerkinElmer, Massachusetts, USA) using attenuated total reflection. Samples of as synthesised PUDs (Table S1) were prepared as thin films by drying on the flat surface of the ATR attachment for 48 hours at room temperature, to eliminate the signal arising from the strong absorption of water.

PSC Model Fitting

The fractional surface area coverage of carboxyl groups (SA_{frac}) with respect to SS M_n was obtained from fitting of the particle surface charge (PSC) model adapted for PUDs (Equations 11 -16) (Table S4).

Table S5. Fractional surface area coverage of carboxyl groups required for stable spherical particles (SA_{frac}) with respect to soft segment M_n from fitting of the PSC model adapted for PUDs.

**Soft segment M_n /gmol^{-1}**	**SA_{frac}**
650	0.107
1000	0.147
2000	0.262

References

(1) Nečas, D.; Klapetek, P. Gwyddion: An open-source software for SPM data analysis. *Central European Journal of Physics* **2012**, *10* (1), 181-188. DOI: 10.2478/S11534-011-0096-2.
(2) Ilavsky, J.; Jemian, P. R. Irena: Tool suite for modeling and analysis of small-angle scattering. *Journal of Applied Crystallography* **2009**, *42* (2), 347-353. DOI: 10.1107/S0021889809002222.
(3) Kell, S. Density, Thermal Expansivity, and Compressibility of Liquid Water from 0" to 150°C: Correlations and Tables for Atmospheric Pressure and Saturation Reviewed and Expressed on 1968 Temperature Scale. *Journal of Chemical and Engineering Data* **1975**, *2* (0), 97-105. DOI: 10.1021/je60064a005.
(4) Pedersen, J. S.; Hamley, I. W.; Ryu, C. Y.; Lodge, T. P. Contrast variation small-angle neutron scattering study of the structure of block copolymer micelles in a slightly selective solvent at semidilute concentrations. *Macromolecules* **2000**, *33* (2), 542-550. DOI: 10.1021/ma990740w.
(5) Register, R. A.; Pruckmayr, G.; Cooper, S. L. Effect of Ionic Aggregation on Ionomer Chain Dimensions. 2. Sulfonated Polyurethanes. *Macromolecules* **1990**, *23*, 3023-3026. DOI: 10.1021/ma00213a035.
(6) Aguiar, R.; Petel, O. E.; Miller, R. E. Effect of a Halloysite-polyurethane nanocomposite interlayer on the ballistic performance of laminate transparent armour. *Composites Part C: Open Access* **2022**, *7*, 100231-100231. DOI: 10.1016/J.JCOMC.2022.100231.
(7) Kašpárková, V.; Šimek, L.; Bohdanecký, M. Estimation of the conformational characteristics of polyurethane chains. *Macromolecular Chemistry and Physics* **1996**, *197* (11), 3757-3772. DOI: 10.1002/MACP.1996.021971121.
(8) Yılgo, S.; Yurtsever, E.; Erman, B. Conformational Analysis of Model Poly(ether urethane) Chains in the Unperturbed State and under External Forces. *Macromolecules* **2002**, *35* (26), 9825-9831. DOI: 10.1021/ma021217b.
(9) Evans, M.; Hastings, N.; Peacock, B. *Statistical Distributions*; John Wiley and Sons Inc., 2000.
(10) Allen, A. J.; Krueger, S.; Skandan, G.; Long, G. G.; Hahn, H.; Kerch, H. M.; Parker, J. C.; All, M. N. Microstructural Evolution during the Sintering of Nanostructured Ceramic Oxides. *Journal of the American Ceramic Society* **1996**, *79* (5), 1201-1212. DOI: 10.1111/j.1151-2916.1996.tb08573.x.
(11) Herdan, G. *Small Particle Statistics*; Butterworths, 1960.
(12) Racine, J. S. RStudio: A Platform‐Independent IDE for R and Sweave. *Journal of Applied Econometrics* **2012**, *27* (1), 167-172. DOI: 10.1002/jae.1278.
